# Integration of Machine Learning and Coarse-Grained Molecular Simulations for Polymer Materials: Physical Understandings and Molecular Design

**DOI:** 10.3389/fchem.2021.820417

**Published:** 2022-01-24

**Authors:** Danh Nguyen, Lei Tao, Ying Li

**Affiliations:** ^1^ Department of Mechanical Engineering, University of Connecticut, Mansfield, CT, United States; ^2^ Polymer Program, Institute of Materials Science, University of Connecticut, Mansfield, CT, United States

**Keywords:** sequence-defined polymer, polymer chain, machine learning, coarse-grained molecular dynamics, copolymer, configuration characterization, feed-forward property prediction, inverse molecular design

## Abstract

In recent years, the synthesis of monomer sequence-defined polymers has expanded into broad-spectrum applications in biomedical, chemical, and materials science fields. Pursuing the characterization and inverse design of these polymer systems requires our fundamental understanding not only at the individual monomer level, but also considering the chain scales, such as polymer configuration, self-assembly, and phase separation. However, our accessibility to this field is still rudimentary due to the limitations of traditional design approaches, the complexity of chemical space along with the burdened cost and time issues that prevent us from unveiling the underlying monomer sequence-structure-property relationships. Fortunately, thanks to the recent advancements in molecular dynamics simulations and machine learning (ML) algorithms, the bottlenecks in the tasks of establishing the structure-function correlation of the polymer chains can be overcome. In this review, we will discuss the applications of the integration between ML techniques and coarse-grained molecular dynamics (CGMD) simulations to solve the current issues in polymer science at the chain level. In particular, we focus on the case studies in three important topics—polymeric configuration characterization, feed-forward property prediction, and inverse design—in which CGMD simulations are leveraged to generate training datasets to develop ML-based surrogate models for specific polymer systems and designs. By doing so, this computational hybridization allows us to well establish the monomer sequence-functional behavior relationship of the polymers as well as guide us toward the best polymer chain candidates for the inverse design in undiscovered chemical space with reasonable computational cost and time. Even though there are still limitations and challenges ahead in this field, we finally conclude that this CGMD/ML integration is very promising, not only in the attempt of bridging the monomeric and macroscopic characterizations of polymer materials, but also enabling further tailored designs for sequence-specific polymers with superior properties in many practical applications.

## 1 Introduction

Polymer materials, a class of natural or synthetic substances composed of long-chain molecules, are prevalent, ranging from proteins, cellulose, nucleic acids in a living organism to familiar man-made materials such as concrete, glass, paper, plastics, and rubbers (Council, 1994; Brinson and Catherine Brinson, 2008; Sawyer et al., 2008; Namazi, 2017). A polymeric structure is composed of multiple simpler chemical units, so-called monomers, which are covalently bonded together to form a long-chain macromolecule. The chemical structure of the monomers, as well as their arrangements, govern the properties of polymer from microstructures to physical and mechanical behaviors (Lutz et al., 2013; Lutz et al., 2016; Soroush et al., 2019; Balasubramanian et al., 2021), for instance, conductivity, elasticity, rigidity, or biodegradability can be finely calibrated by the sequence-defined polymers (Hartmann and Börner, 2009; Lutz et al., 2013; Porel and Alabi, 2014; Perry and Sing, 2020).

**FIGURE 1:**
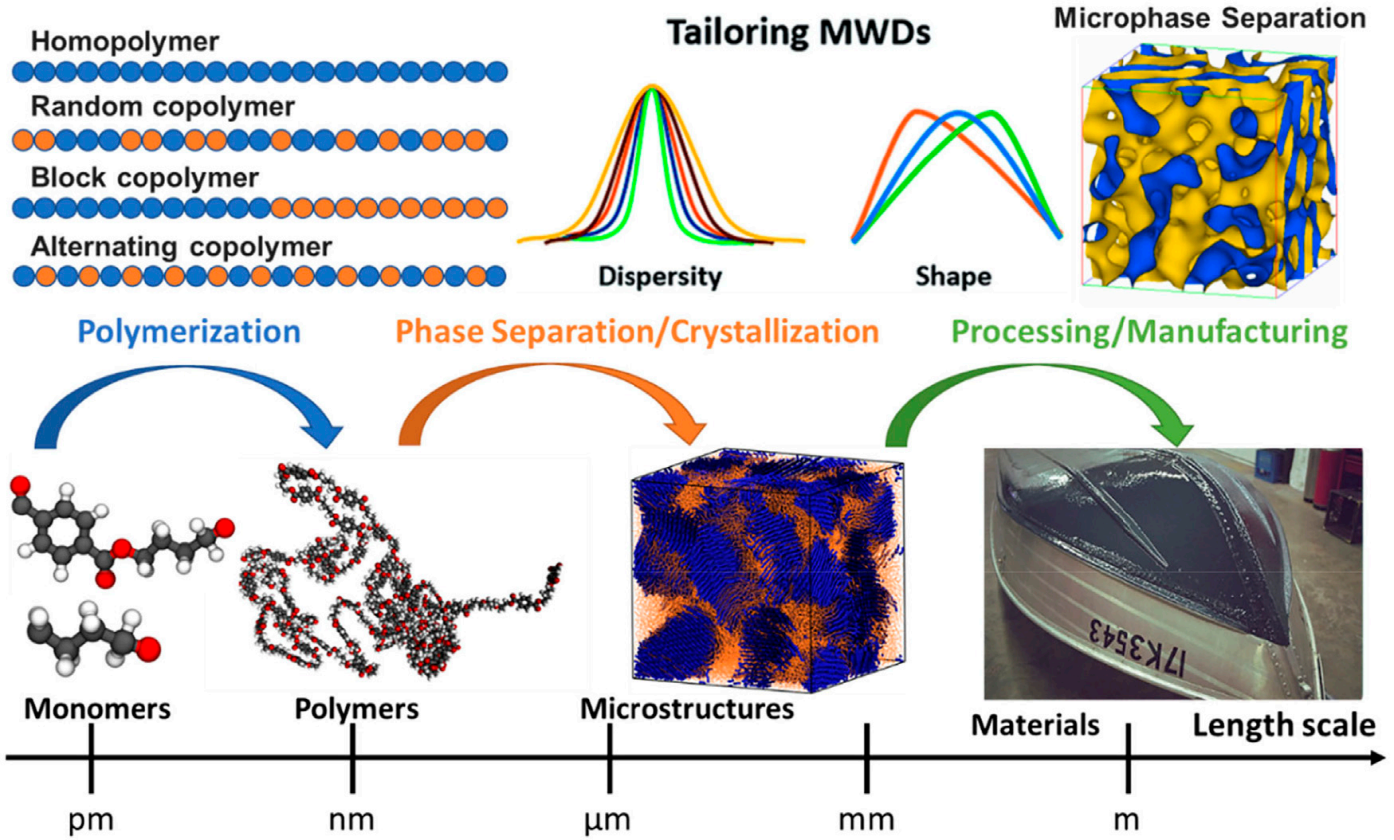
A hierarchical design principle for polymeric materials, considering their monomer, chain, microstructure, processing, and manufacturing. The architecture of polymer chains, along with their molecular compositions (monomers), determines the microstructure of polymers, such as phase separation and crystallization. Monomer is a repeat unit comprising a polymer’s chemical structure. If there is only a single type of monomer, the polymer is known as a homopolymer, while a polymer containing two or more types of monomers is called a copolymer. In copolymers, monomers can arrange in a variety of manners such as random, block, gradient, or alternating. A controlled monomer sequence vigorously influences the polymer chain properties, ultimately leading to the desired phase separation or crystallization of microstructure.

In general, the design of a polymer can be separated into three parts corresponding to three steps in the processing of polymers, as illustrated in Figure 1: molecular design of monomers for polymerization, microstructure formation due to phase separation or crystallization, polymer processing, and manufacturing. Monomer, the building block of polymers, forms the repeating unit of polymers to influence the fundamental physical properties of eventually produced polymers. While molecular weight is one of the critical factors that influence the properties of small organic molecules, the polymer’s size effect is different from its monomer size. Since the polymers are long-chain molecules, their size effect typically originates from the molecular weight rather than the monomer size. Indeed, during the various polymerization process, the molecular weight distribution (MWD) can be formed and tailored through a controlled fashion from the same type of monomer (Gentekos et al., 2016), resulting in significantly different physical and chemical properties (Nunes et al., 1982; Imrie et al., 1994; Gentekos et al., 2019). Hence, various metrics based on the MWD of polymers are used to characterize their sizes to polymer properties (Bur and Fetters, 1976; Colby et al., 1987; Fetters et al., 1994). Furthermore, the inter- and intra-molecular interactions between polymer chains can lead to very different microstructures, such as phase separation and crystallization, influencing their thermal and mechanical properties dramatically (Strawhecker et al., 2013; Hsieh et al., 2014; Yi et al., 2018). Eventually, the same type of polymer can undergo different processing or manufacturing conditions, such as stretching, compressing, or mixing additives, to further enhance or tailor their properties for specific applications (Vasile and Pascu, 2005). Therefore, the design space of polymers should cover all the parameters involved in these steps, such as the molecular space of single or multiple monomers for homopolymers or copolymers respectively, temperature, pressure, polymerization process, molding methods, additives or fillers, etc.

Over the last decade, the development of synthetic polymers focusing on controlling sequence-specific chains has become notable in polymer science and engineering (Badi and Lutz, 2009; Hartmann and Börner, 2009; Chan-Seng et al., 2012; Lutz et al., 2013; Lutz, 2017; Guseva et al., 2017; Solleder et al., 2017; Nanjan and Porel, 2019; DeStefano et al., 2021). Monomer sequence can dictate important properties of the polymer chains, such as polymer configurations, gyration radius, self-assembly behaviors, etc. via the inter- and intra-molecular interactions. Compared to classical random and block copolymers, these sequence-defined polymers provide enormous opportunities for materials design, with tailored microstructure and mechanical properties (Leibfarth et al., 2015; Meier and Barner-Kowollik, 2019; Nanjan and Porel, 2019). However, it also leads to questions that polymer mechanics must address, including but not limited to: 1) To what extend does chain sequence matter for mechanical properties of polymers? Is it worth overcoming the challenge of synthesizing sequence-defined polymers for unique materials design? 2) How does the monomer sequence scale relate to the chain length scale of polymers? Are there fundamental limits associated with how the monomer sequence can determine the structure/mechanics of polymers and vice versa? 3) How do we consider a vast sequence parameter space of sequence-defined polymers? To answer these questions, it is inevitable to boost our fundamental understandings of sequence-structure-property relationships for polymeric materials with not only a single one, but an assemble of polymer chains. Recently, the computational approach has been an effective alternative tool to enhance our predictive capability due to the limitations of current experimental measurements (Binder, 1995; Li et al., 2012a; Li et al., 2013; Li et al., 2017; DeStefano et al., 2021). In particular, molecular dynamics (MD) simulation has demonstrated its robustness in capturing physical and mechanical properties of polymers, such as glass transition temperature (Varnik et al., 2002), viscosity (Mondello and Grest, 1997), dynamics and relaxation (Binder, 1995), phase separation (Tanaka, 1993), crystalization (Kavassalis and Sundararajan, 1993; Gee et al., 2006), entanglement network (Everaers et al., 2004; Kröger, 2005), Young’s modulus and yield strength (Li and Strachan, 2011). Among different MD techniques, coarse-grained molecular dynamics (CGMD) rather than all-atom modeling can serve as an effective approach for reducing tremendously computational cost and complexity of chemical space while maintaining modeling accuracy (Li et al., 2013; Ingólfsson et al., 2014; Webb et al., 2019; Wang et al., 2020a; Ye et al., 2021). Nevertheless, carrying out the CGMD simulations for all potential candidates is impossible because this would be extremely computationally demanding and time-consuming, limiting the CGMD applications in the design and discovery of new polymers. For instance, if the polymer chain is composed of ten of two types of monomers, the number of possible chains for sampling is around 500, which is still feasible. Nonetheless, if increasing the length to 30, the possibilities increase exponentially to more than 500 million (Patra et al., 2020).

Using theory in combination with MD simulations is a conventional approach to boost the simulations in polymeric systems. Different from MD simulations in which the coordinates (including bond and torsional angles) of the atoms or particles will be solved, theory-based simulations focus on the functional integrals over the chemical potential fields in a simulation domain. Thus, a discrete bead-spring chain model from MD simulations can be simplified by a continuous chain (a space curve) (Fredrickson et al., 2002). We can visualize the combination between MD simulations and theoretical approach in which MD simulations can be used to provide structural information into or validate the theory-based method, and then the theoretical model is used to explore the polymeric properties in larger parameter space, time, and length scales that are too computationally expensive when using MD simulations alone. This combination allows us to overcome the limitations of MD simulations, such as the size effects of finite systems, conformational transitions with large length and time scales, or long polymer relaxation times (Gartner and Jayaraman, 2019). Common theories used in polymer simulations include field-theoretic computer simulation (FTCS), self-consistent-field theory (SCFT)/density functional theory (DFT), dynamic mean-field theory (DMFT), integral equation polymer reference interaction site model (PRISM). Each of these methods has advantages and disadvantages. For instance, FTCS can work well in concentrated systems of high molecular weight polymers (e.g., dense polymer solutions, molten blends, block copolymers, and their composites) as well as systems with soft, long-ranged interactions (e.g., electrolyte solutions, polyelectrolytes, block co-polyelectrolytes), but turned out to be inaccurate in dilute and semi-dilute polymer solutions (Fredrickson et al., 2002). SCFT/DFT can work efficiently with perplexing architecture copolymers in bulk and self-assembled structures in dilute copolymer solutions (Wang et al., 2018), but the method accuracy might be vulnerable to system fluctuations (Zhang et al., 2007). Classical DFT shares a number of similarities with the polymer SCFT and can reproduce the morphologies of block copolymer thin films predicted by SCFT as well as resolve the structural properties near the interface, but needs more development for complex systems with multidimensional density profiles (Frischknecht et al., 2002). Another approach is the DMFT which proved to be effective to simulate processes with length and time scales for the polymer systems currently inaccessible by MD simulations (Knoll et al., 2004), but inefficient for nonlocal coupling effect due to the huge computational expense to acquire the chemical potential (Zhang et al., 2017). One interesting approach that has recently received much attention is the use of PRISM. PRISM theory describes the liquid-like structural correlations in single- and multicomponent polymer melts, solutions, nanocomposites, and complex fluid systems, and the theory has been validated for many polymeric systems (Martin et al., 2018). The theory uses the “closure relations” (intra- and inter-molecular correlation functions) to reflect the pairwise interaction potentials acting between components. The integration of MD and PRISM theory can be done by using the physical and chemical features of the MD models to import into the PRISM model and numerically solving the PRISM theory. The qualitative and quantitative agreements between PRISM and MD simulation results in the study of copolymers show that PRISM theory can be used as an effective tool to guide MD simulations and experiments in the study of block copolymer assemblies with various copolymer sequences and compositions (Lyubimov et al., 2017). Additionally, since PRISM calculations are much faster than all-atomistic MD simulations, it allows the exploration of a much larger parameter space such as different block copolymer architectures, sequences, compositions, etc. However, this theory-based method also has some limitations. PRISM cannot be directly applied for the structure of ordered, macrophase-separated, or microphase-separated materials that are different from the liquid-like system. Additionally, the theory produces only pair correlation functions rather than a coordinate trajectory, thus leading to the lack of visual analysis. The method can be extremely slow to converge and unstable in some conditions (Martin et al., 2018).

When it comes to the issue of big data, artificial intelligence (AI) or machine learning (ML) is considered one of the best computational tools for problem-solving. ML is enabled by preexisting experimental and/or computational data and is extremely useful not only for polymer applications but for materials discovery and characterizations (Hill et al., 2016; Liu et al., 2017; Ramprasad et al., 2017; Butler et al., 2018; Chen et al., 2020; Lopez-Bezanilla and Littlewood, 2020; Saal et al., 2020; Chen et al., 2021a; Batra et al., 2021). However, most of the current training data for ML algorithms in polymer applications are derived from the DFT calculations of monomeric or small oligomeric substances (Webb et al., 2020). Additionally, the polymeric structures used in the current ML models are mostly represented by a simplified molecular-input line-entry system (SMILES) of the monomers for simplification (Chandrasekaran et al., 2020; Zhu et al., 2020; Nazarova et al., 2021). We know that SMILES is one of the most popular methods to represent molecules because it is handy and readable for both humans and machines. Even though the SMILES is applicable in homopolymer property predictions, the influence of polymer chain topology on the target properties is almost excluded (Pilania et al., 2019; Deacy et al., 2021; Patel et al., 2021). It seems ineffective to obtain understandings of the macromolecular behaviors such as self-assembly, polymeric crystallization or knot-type classifications, etc. when the polymer chain is represented by its monomer only. Therefore, using the traditional ML approaches with the current polymeric database and monomeric representation might not be sufficient to comprehensively acquire the macroscopic behaviors of polymers. To overcome this challenge, researchers have been integrating CGMD with ML tools to effectively accelerate the polymer chain and microstructure design (Jackson et al., 2019; Webb et al., 2020; Arora et al., 2021; Jablonka et al., 2021; Schneider and de Pablo, 2021). In this way, the CGMD simulation is used to generate the training datasets for polymer chains (configurations and/or microstructures), and then ML algorithms are implemented to establish surrogate models for polymer chain characterizations or inverse designs. ML is very powerful since it does not require a rigorous theoretical description of polymeric materials and, more importantly, can handle sparse training data (Zhang and Ling, 2018). This is very helpful under some difficult or labor-intensive circumstances for measuring or sampling, for instance, MD simulation for a very large system. Therefore, in the current lacking of clear physics-based models and polymer datasets, the hybridization of ML and CGMD (CGMD/ML) allows us to speed up the problem-solving at the scale of the polymer chain or network, particularly outside the “comfort zone” of these materials. However, like other computational approaches, ML tools in the field of polymers also face some limitations that need to take into account in the future, including 1) Dataset availability; 2) Feature representations and 3) Transferability. Compared to the more experienced bioinformatics or material informatics databases, there are fewer databases for polymeric materials, especially at the chain-length level. ML algorithms at this level require a sufficient number of data points derived from MD simulations; thus when it comes to an unexplored corner of polymer material (or novel structures), one needs to generate the MD simulation dataset, which might take a lot of time and computational cost to acquire and process the raw data. Therefore, it will be encouraged to obtain a constitution and variety of such databases to derive maximum utility for the polymer materials community. Another concern is the choice of the feature representations. Efficient descriptors are considered to be 1) invariant to the symmetries of the underlying physics; 2) easy to interpret; 3) expressed in a direct and concise form and 4) computationally efficient (Haghighatlari and Hachmann, 2019). However, it is still challenging to develop descriptors that could satisfy all these criteria. Thus, it requires more effort to standardize the feature representation and selection for ML models in the future. Another biggest question is related to the accuracy and transferability of the ML models. For instance, even though one can use an ML algorithm to tackle a specific CG polymer system, but it does not guarantee this ML model can be applicable in a new CG polymer setup with different coarse-graining representations. Therefore, making a better transferable ML model also requires further ML algorithm development as well as a suitable coarse-graining mapping for polymer systems.

Although several excellent reviews already exist in the application of ML algorithms in the field of polymers and soft matters (Audus and de Pablo, 2017; Ramprasad et al., 2017; Zhang and Ling, 2018; Jackson et al., 2019; Chen et al., 2020; Batra et al., 2021; Clegg, 2021; Deacy et al., 2021; Friederich et al., 2021; Sattari et al., 2021; Sha et al., 2021), none of them thoroughly focused on studying the combination of ML algorithms and CGMD modeling of polymer chains. Therefore, we aim to promote our understanding of this fascinating topic by providing case studies on the integration of these two powerful computational techniques in polymer science. To this end, this review is organized as follows: Section 2 provides basis description of MD simulations for polymer system; Section 3 describes an overview of ML approaches with essential knowledge and then provides some commonly used ML algorithms in polymeric materials; Section 4 introduces the most recent CGMD/ML studies related to the three most common polymer chain applications including configuration classification, feed-forward property prediction, and inverse design; Section 5 highlights main challenges and future directions expected to go in this field. Finally, Section 6 presents our summary and conclusions of the promising employment of CGMD/ML in polymer chain studies.

## 2 Basic Description of MD Simulations for Polymers

Due to the limitations of current experimental techniques, molecular dynamic (MD) simulations have provided an effective alternative approach to characterize the structures and molecular mechanisms of polymers. In MD simulations, the equations of motion are used to derive the positions and velocities of atoms and molecules via external and interatomic forces (Brighenti et al., 2020). By doing so, we are able to explore the molecular structures and thermodynamic properties of polymers. The emergence of MD simulations not only helps to understand the experimental observation but also significantly assists the molecular and macroscopic modeling.

### 2.1 The Spatial and Temporal Scales of Polymer Simulations

**FIGURE 2:**
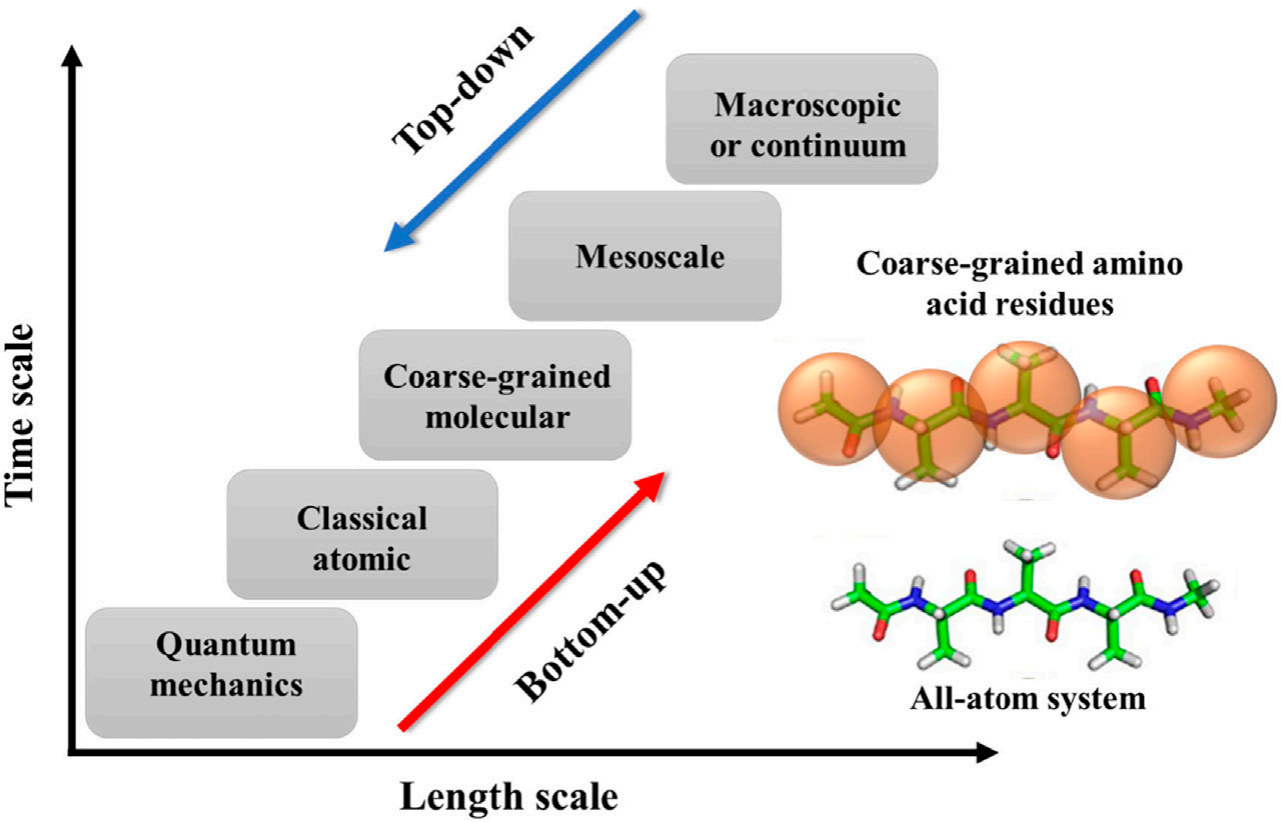
Schematic diagram to show different spatial and temporal scales in the MD simulations of polymers as well as two types of coarse-grained methods: bottom-up and top-down. An example of the coarse-graining process of a polypeptide (polyalanine) is shown in the inset. Figure was reproduced from Ref. (Ye et al., 2021) with permission.

The simulations for polymers can have various temporal and spatial scales, including quantum mechanics, atomistic scale, coarse-grained (CG)/mesoscopic scale, and macroscale (Figure 2). Briefly, the quantum scale is about 10^−10^ m and 10^−12^ s. The particles at this scale include the nuclei and electrons, and their configurations are solved using quantum mechanics. At the atomistic scale (∼10^−9^ m and ∼10^−9^–10^−6^ s), all-atoms (AA) models are explicitly represented by their nuclei as single sites. The interactions between atoms include bonded and non-bonded forces. The former interaction accounts for bond length, bond angle, and bond dihedral potentials. The latter one usually uses Coulomb interactions and dispersion forces. At the coarse-grained and mesoscopic scale (∼10^−6^ m and ∼10^−6^–10^−3^ s), a molecule of polymer will be represented by a number of microscopic particles known as a bead. This coarse level of modeling allows us to simulate the polymer system with larger spatial and temporal scales than the previous models. The last scale is called the macroscopic or continuum scale that is in the order of 10^−3^ m and 1 s. At this level of modeling, the polymer system is described as a continuous medium without discrete atoms and molecules. The model uses constitutive and conservation laws to capture the macroscopic phenomena in polymeric systems that are typically acquired via experimental measurements. Since coarse-grained molecular dynamics (CGMD) simulation is one of the significant computational approaches of this review, we are going to provide more detail about it.

### 2.2 Basic Description of CGMD Simulation for Polymers

CGMD is a process of reducing atomistic systems with fewer degrees of freedom in order to simplify the polymeric system. The models consist of CG beads corresponding to a group of atoms of the polymers, thus leading to fewer degrees of freedom to compute as well as neighbors to take into account per particle. By doing so, the CGMD can reduce the complexity of the system, increase the computational efficiency while retaining several important properties of MD simulations at the atomistic level, and allow a longer temporal scale. The CGMD usually includes two key steps: 1) mapping CG beads from the AA scale model to reduce the complexity of the system; and 2) defining the interaction between these CG beads.

#### 2.2.1 Mapping the CG Beads

The mapping procedures aim to represent small groups of atoms from all-atom (AA) simulation with CG beads (one example of a polypeptide shown in Figure 2). The number of heavy atoms represented by a CG bead reflects the degree of coarse-graining. Besides the coarse-graining level, the mapping must take into account the important physics and chemistry of the polymer system as well (Dallavalle and van der Vegt, 2017). Additionally, in the presence of a solvent, it is essential that CG models also consider solvent-solvent and solvent-solute interactions to reproduce the experimental observations, especially for biological and thermosensitive polymers (Joshi and Deshmukh, 2021). The chosen mapping scheme must also guarantee the statistical correlations of internal degrees of freedom so that we can distinguish the bonded interactions with the bond (stretching), angle (bending), and dihedral (torsion) terms (Peter et al., 2008). Typically, the centers of CG beads are determined such that the connection between these beads can be expressed by a single harmonic potential (Abrams and Kremer, 2002).

#### 2.2.2 Defining the Interactions Between CG Beads

Once we have the CG representation of the polymeric molecule, we need to define the interactions between the CG beads. This process includes two different approaches: bottom-up and top-down (Figure 2). In the bottom-up approach: we adopt the AA simulation (as a reference) to derive the force fields or interactions between CG beads. The microscopic thermodynamics and structural properties derived from AA modeling will be used to calibrate the force fields of CG beads. However, this approach is not transferable from one system to another due to its specific AA representation. Another method that can be more generic is called the top-down approach. In this approach, the force field of CG beads is tuned from macroscopic experimental observation, which exhibits a great ability of transferability. The interactions between beads can be either generic or chemistry-specific. In the generic force field, the beads are lack chemical information for specific systems. The generic models employ potentials with fewer parameters but can be efficiently used to investigate the influence of molecular parameters on different properties (Cooke et al., 2005). Among generic force fields, finite extensible nonlinear elastic (FENE) (Li et al., 2012b) is a common “bead-spring” CG model used in many polymeric studies. On the other hand, the chemically specific models use the multi-property fitting approach to parameterize the potentials and replicate the AA model’s observations (Shinoda et al., 2007). One of the most common ones in chemical-defined models is the Martini force fields for polymer and biological systems (Marrink and Tieleman, 2013). The Martini model follows a four-to-one mapping that means, on average, a single CG bead represents four heavy atoms. Based on the chemical nature of the polymer structure, the CG beads are assigned a specific particle type with more or less polar properties, including polar (P), non-polar (N), apolar (C), and charged (Q). Within each bead type, there are also subtypes with specific chemical properties giving a total of 18 different “building blocks”. In the Martini models, the non-bonded interactions are parameterized based on experimental thermodynamic data, while the bonded interactions are tuned to reproduce the AA simulation results (Marrink and Tieleman, 2013). The Martini can reduce a lot of chemical complexity with stable simulations in a variety of applications in polymer and biological fields.

In MD simulations for polymers, the interactions between CG beads include bonded and non-bonded interactions. The bonded interactions typically include bonds, angles, dihedrals, and impropers. The bond potentials are commonly used with the empirical form of finite extensible nonlinear elastic (FENE) potential. The form of FENE potential is as follow (Li et al., 2013):

$$V_{FENE}\left(r\right)=\;-\frac{1}{2}KR_{0}^{2}\;ln\left[1-{\left(\frac{r}{R_{0}}\right)}^{2}\right],$$ (1)

where *K* is the bond strength (usually *K* = 30, to avoid bond crossing) and *R*_*0*_ = 1.5σ is used as the maximum bond length. Another common generic form of bonded potential is the harmonic bond force as follow:

$$U_{bond}=K{\left(r-r_{0}\right)}^{2},$$ (2)

in which *K* is the bonded force constant, and *r*_*0*_ is the equilibrium bond length. The angle is typically used with a harmonic angle potential. This potential has the form:

$$U_{angle}=K_{\theta }{\left(\theta -\theta_{0}\right)}^{2},$$ (3)

where K_*θ*_ is the angle force constant and *θ*_*0*_ is the equilibrium angle. The dihedrals and impropers are used for torsion potentials based on the quartet of CG beads. The cosine and harmonic potentials are the most commonly used for these two interactions. These generic potential functions can reduce the computational time while fairly maintaining the polymeric molecular structures.

Non-bonded interactions account for the attractive and repulsive forces between CG beads, namely Van der Waals and electrostatic forces. The non-bonded interactions determine the macroscopic properties in soft matter systems (Peter and Kremer, 2009). The electrostatic interaction between two beads can be expressed with Coulomb’s law:

$$U_{electrostatic}=\;\frac{q_{i}q_{j}}{4\pi \varepsilon_{0}\varepsilon_{R}r},$$ (4)

where *q*_*i*_ and *q*_*j*_ are the charges of two interacting beads, *ε*_*0*_ is the permittivity of vacuum, *ε*_*R*_ is the dielectric constant, and *r* is the distance between interacting beads. The Van der Waals interactions are commonly used with Lennard-Jones (LJ) 12-6 potential in the following form:

$$U_{LJ}=\;\;4\varepsilon \left[{\left(\frac{\sigma }{r}\right)}^{12}-{\left(\frac{\sigma }{r}\right)}^{6}\right],$$ (5)

where *ε* is the depth of the potential well, *σ* is the collision diameter, and *r* is the distance between the particles. The LJ potential includes repulsion short-range and attractive long-range terms (Wang et al., 2020b).

Another coarse-grained modeling approach for polymer systems that is different from the aforementioned methods is called the mesoscopic particle-based model or dissipative particle dynamics (DPD). This is a simulation technique developed for Newtonian and non-Newtonian fluids. In this method, the elementary unit is not an atom or molecule, but a collection of atoms. In DPD, two particles *i* and *j* interact with a sum of different forces, including conservative $\left(F_{ij}^{C}\right)$ , dissipative $\left(F_{ij}^{D}\right)$ and random forces $\left(F_{ij}^{R}\right)$ :

$$F_{ij}^{DPD}=\;F_{ij}^{C}+F_{ij}^{D}+F_{ij}^{R}.,$$ (6)

For polymer simulation, the DPD method considers polymers as a chain of soft CG beads, and each CG bead represents a group of monomers in a whole polymer structure. In DPD, the CG spheres interact with each other through purely repulsive soft potentials. These interactions between beads can be fine-tuned to capture the macroscopic phenomena on larger time scales. The approach is more effective in studying the mesoscale properties, such as the flow of polymer fluids and the growth of self-assembled morphologies (Wang et al., 2021a).

We have known that the force fields or interactions between CG beads are crucial to capture the microscopic and macroscopic behaviors of polymer systems. The question is, how can we acquire these interaction parameters? The answer comes from the force-field parameterizing (FFP) process. One of the most widely used FFP approaches is the Iterative Boltzmann Inversion (IBI) method (Moore et al., 2014). It is used to determine the bead-bead interactions which match the structural properties (the radial distribution function or RDF) from the AA simulation reference. In practice, the probability distribution function for the AA model, *p*_R_, can be estimated directly from trajectories of MD simulations and considered to depend on the following four variables: pair distance (*r*), bond length (*l*), bond angle (*θ*), and dihedral angle (*ψ*). The potential function for the corresponding CG system is determined through the following equation (Li et al., 2013):

$$U\left(R^{N}\right)=\;-k_{B}T\ln p_{R}\left(R^{N}\right).$$ (7)

If we assume the above four variables are independent of each other, then the potential function for the coarse-grained model becomes:

$$U\left(R^{N}\right)=U\left(r;l;\theta ;\psi \right)=U\left(r\right)+U\left(l\right)+U\left(\theta \right)+U\left(\psi \right),$$ (8)

where *U* (*q*) = *−k*_B_ *T* ln *p*_*R*_ (*q*) with *q* = *r*; *l*; *θ*; *ψ* for pair, bond, angle and dihedral interactions, respectively. In order to replicate the distribution function of AA reference, the iterative parameter optimization for CG potential is then implemented as:

$$\begin{array}{c} U^{n+1}\left(q\right)=\;U^{n}\left(q\right)+\Delta U^{n}\left(q\right)\\ \Delta U^{n}\left(q\right)=\;k_{B}T\;\ln\;\frac{p_{R}^{n}\left(q\right)}{p_{R}^{target}\left(q\right)} \end{array},$$ (9)

where *U* ^*n*^ is the CG potential after step *n*, *k*_*B*_ is the Boltzmann constant, *T* is the absolute temperature, $p_{R}^{target}$ are the target distribution functions calculated from the all-atomistic molecular simulations. Thus, the distribution functions, *p*_*R*_, can converge to the target distribution functions, $p_{R}^{target}$ , after several iterations. The IBI is a structure-based parameterizing method. Another common method is called force-based procedure, which is based on the matching of force distributions from AA simulations to CG beads. This approach includes force matching, multiscale coarse-graining (MS-CG), stochastic parametric optimization (SPO), relative entropy minimization, etc. The aforementioned techniques are traditional ones. With the aid of more advanced optimization algorithms and machine learning, the force field parameterizing process can now be achieved much faster with higher accuracy (Ye et al., 2021).

### 2.3 Selecting an Ensemble or Thermostat

After defining the CG mapping, the force field potentials, and the structure of the polymer, the next important step is to choose a suitable thermostat or ensemble. There are multiple options for selecting the type of thermodynamics ensemble to sample depending on the purpose of users as well as experimental conditions. The microcanonical statistical-mechanical ensemble or NVE is a common one where the number of atoms (N), volume (V), and total energy (E) of the simulation box are maintained constant. The simplest extension of the NVE ensemble is the isothermal-isochoric or NVT, in which the kinetic energy of the system at a specific temperature remains constant. Other common approaches are the isothermal–isobaric (NPT) with constant pressure P and grand canonical (*μ*VT) with constant chemical potential *μ* as well as the isothermal-isostress (NσT) ensembles. Among those, NPT simulations are preferable because this ensemble is mostly comparable to experimental conditions (constant pressure and temperature condition are doable in the lab environments), yet the simulation box size needs to be taken into account during the simulations. The most common thermostats used in MD simulation for polymer systems include the velocity Verlet algorithms, the Nose-Hoover thermostat, and stochastic thermostat or Brownian dynamics.

## 3 Machine Learning Algorithms

### 3.1 Overview of Machine Learning Methods

**FIGURE 3:**
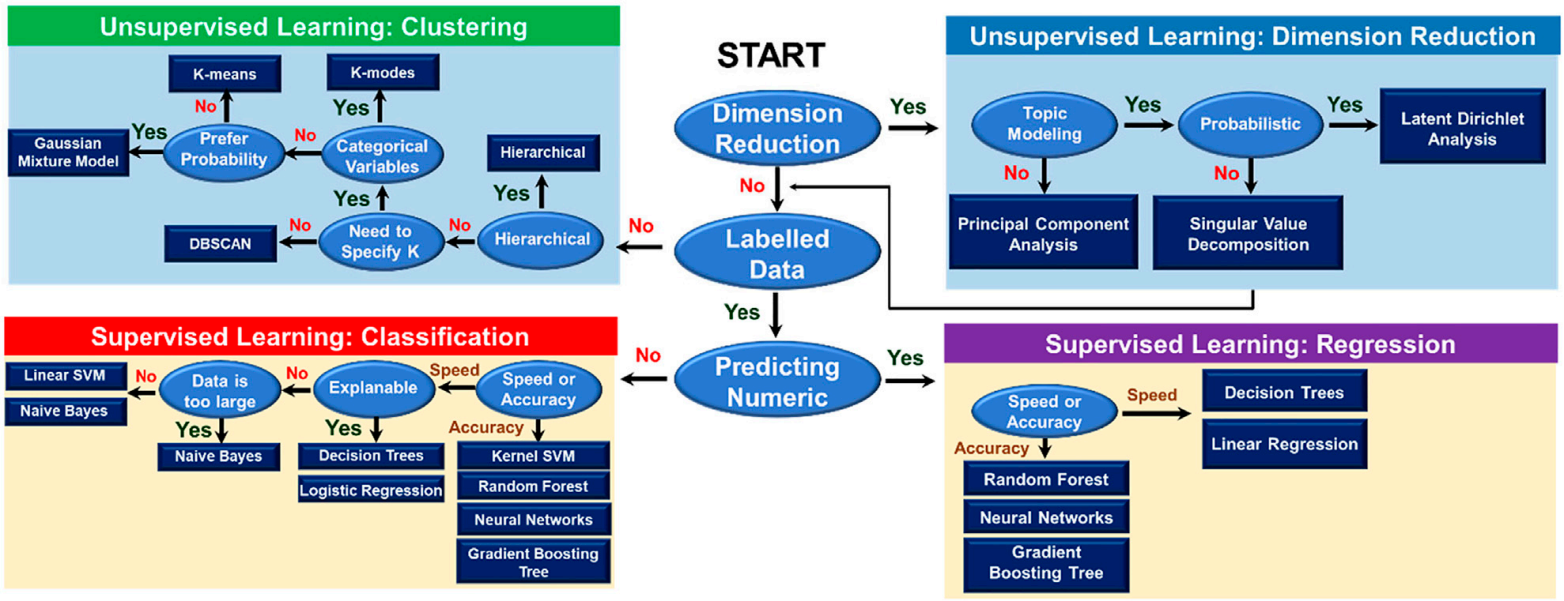
Overview of machine learning algorithms. The flowchart guides users in the selection of an appropriate machine learning algorithm. There are two main types of learning, including supervised and unsupervised learning. The selection of relevant algorithms in each type of learning method depends on the purposes of learning, such as clustering, dimension reduction, classification, or regression.

ML involves a broad field of artificial intelligence, computer science, data analysis, and every individual branch in ML constitutes an area of research. Generally, ML is utilized to identify patterns from data and make decisions accordingly. There are two basic types of learning that are usually formulated, including supervised and unsupervised learning (Figure 3). In supervised learning, previously collected data is required to train ML models. The training data with corresponding results, namely the labeled data, is necessary in this case. Trained with labeled data, the supervised ML model learns the pattern that maps input data to output results. Unsupervised learning, meanwhile, not requiring the labeled data, directly learns the pattern of the data and differentiates data points into clusters with similar features. As shown in Figure 3, for each type of learning, the selection of relevant algorithms depends on the purpose of users, such as classification or regression in supervised learning and clustering or dimension reduction in unsupervised learning. Besides two main types, there are semi-supervised and reinforcement learning. Combining supervised learning and unsupervised learning brings out semi-supervised learning. In semi-supervised learning, active learning is a technique recently getting more attention, especially in inverse designs due to its ability to sort out what data should be collected or used for the model training (Lookman et al., 2019). It is normally applied in cases where obtaining labels is expensive (either computationally or experimentally), so the model defines a strategy to maximize the usefulness of the new data point. Last but not least, reinforcement learning is a method to force an agent to learn how to make decisions based on feedback from its environment (Palminteri et al., 2013). This type of learning is one of the most researched fields in ML. Such as in game theory, reinforcement learning can guide players to maximize their score by finding the optimal solution to each movement (Singh et al., 2017). The choice of ML method is usually problem or application-dependent. In the later parts, we are going to discuss some of the ML algorithms that are more commonly used in the field of polymer informatics, particularly at the scale of the polymer chain.

### 3.2 Feed-Forward Neural Networks

Feed-forward Neural Networks (FNNs) model is a type of deep neural network or referred to as multilayer perceptrons. FNNs can be applied in supervised learning classification and regression. Its goal is to approximate a function *y* = *f* (*x*; *θ*) that maps an input *x* to an output *y* where the parameters *θ* define the mapping relation. The FNNs suggest a feed-forward information flow that passes through the function of input *x*, then through the intermediate computations of hidden neurons, and finally to the output *y*. The training of FNNs corresponds to minimizing a loss function, through which the weights and biases in the parameters *θ* are optimized to get an improved mapping performance. Common loss functions include mean square error (MSE), mean absolute error (MAE), and root mean squared error (RMSE), etc. To minimize the loss function, gradient-based (GB) algorithms such as Stochastic Gradient Descent (SGD) and Adaptive Moment Estimation (Adam) are mostly used to search for the optimal parameters (Ruder, 2017). As a branch of FFNs, a deep neural networks (DNNs) model is an improvised neural network with many more layers. Different from traditional ML methods, the DNN algorithm is given raw data and identifies for itself what features are appropriate. It is efficiently used for training large amounts of data and learning more complex patterns (Najafabadi et al., 2015; Becker et al., 2020; Verpoort et al., 2020).

### 3.3 Recurrent Neural Networks

Recurrent Neural Networks (RNNs) extend from FNNs to include feedback connections between layers, such that an extra loop is added to the original feed-forward information flow. RNN is suitable for processing sequential data such as characters or words, such as natural language processing (Cho et al., 2014). It learns a pattern from past tokens and is able to predict the next tokens in a sequence. However, the basic RNNs architecture is known to suffer from a short-term memory issue. If a sequence is very lengthy, it will be difficult to carry information from earlier to later time steps. Therefore, RNNs may leave out important information from the beginning in case of processing a long paragraph to make predictions. Additionally, RNNs suffer from the vanishing gradient problem during backpropagation (Sak et al., 2014). Gradients are essential values to update the weight of neural networks, but the vanishing gradient problem makes the weight updating unachievable. If the gradient shrinks as it back propagates through each time step, the gradient value becomes extremely small, and it will not contribute much to the learning. To deal with the short-term memory and vanishing gradient issues, new RNNs architectures like Long Short-Term Memory (LSTM) or Gated Recurrent Unit (GRU) have been proposed (Dey and Salem, 2017; Sherstinsky, 2020). These additions use gates mechanisms to regulate the information flow through the sequence, and automatically learn to keep only relevant information and forget non-relevant information to make predictions.

### 3.4 Convolutional Neural Networks

Convolutional Neural Networks (CNNs) represent another type of FNNs. Instead of only using fully connected layers, CNNs model contains special convolutional layers that are particularly designed to extract features from image inputs (Liu, 2018). In a convolutional layer, one three-dimensional (3D) filter matrix converts a volume of neurons in the previous layer into a new neuron in the current layer, and a set of 3D filters convert a volume of neurons into a new volume of neurons. CNNs models have flexible architectures as the filter size, and sliding step is arbitrary. One challenge is that convolutional layers based on multiple filter matrices may have too many parameters to optimize. Overfitting happens easily when the model is so complex while the training data is limited. Therefore, a pooling layer is usually added to reduce the dimension of convolutional layers, consequently, reduce the complexity of the CNNs model. Max pooling is the most commonly used one that extracts the maximum value from the convolved features and passes it to the next layer. Although the convolutional layer plus pooling layer architecture was originally designed to process image input, other lower-dimensional inputs such as one-dimensional (1D) vectors are also feasible for CNNs.

### 3.5 Decision Tree and Random Forest

A decision tree (DT) is a tree-like model where each node represents an observation, and each branch represents the possible consequences. A decision tree can be a classification tree or a regression tree, based on the target variable represented by the leaves. No matter the leaves are discrete values or continuous values, the model can go through each node along with a series of branches and reach the target value. The key to building a DT model is to find the best attribute to test in each decision node, and the model training is an optimization of the tree shape and node arrangement. Each optimization may result in a different tree shape and node arrangement, accompanied by many model uncertainties. To make the model more robust, a special DT-based model-random forest (RF)—is developed with the ensemble method. RF is composed of a set of DT, and their results are combined to make final predictions. It is found that although each tree makes its own prediction, averaging multiple DTs reduces the model variance and generates a more accurate prediction compared to any single DT (Qiu and Fan, 2021).

### 3.6 Gaussian Process Regression

Gaussian process regression (GPR) is a nonparametric model that is function-free in the initial setup. It avoids the optimization of a specific function but calculates the probability distribution of all possible functions that fit the data. The first step in GPR is to specify a prior Gaussian process on the function space, such as the mean and covariance functions. This allows the incorporation of prior knowledge about the functional space. Common covariance kernel functions can be constant, linear, or square exponential. With the Gaussian process prior specified, optimization is then carried out to tune the function hyperparameters using the training data. At last, the obtained posterior compute the predictive distribution on the new data points. Compared to other ML methods, the GPR model also provides uncertainty intervals together with prediction values. This unique feature makes GPR valuable whenever uncertainty estimates are especially demanded (Deringer et al., 2021).

### 3.7 Generative Models

In ML, generating new data from the existing dataset is sometimes necessary in case of a limited source of data for the training process. To overcome it, a generative model (GM) is developed that learns true data distribution from the current training set and then generates new data points with some variations. Among GM algorithms, two major families stand out and deserve special attention: Variational Autoencoders (VAEs) (Kingma and Welling, 2013) and Generative Adversarial Networks (GANs) (Goodfellow, 2014). VAE transforms high dimension data into lower-dimensional latent space through its encoder. The encoding distribution is regularized during the training in order to ensure that its latent space has proper features so that the following decoder can generate similar new data. The encoder produces the “new features” representation from the “old features” representation, and the decoder is the reverse process to reconstruct the data. GAN model, also containing two components, utilizes a generator and a discriminator to play an adversarial game against each other. The generator aims to generate new data (fake ones) while the discriminator tries to identify its authenticity. When GANs are fully optimized, the generated data is so like the true data that the discriminator cannot tell the difference. VAEs and GANs have demonstrated excellent performances in many polymer and materials informatics applications (Elton et al., 2019; Shmilovich et al., 2020; Yang et al., 2021).

### 3.8 Bayesian Optimization

Bayesian optimization (BO) is one of the most common active learning approaches that is recently getting much attention in polymers and materials design (Hou et al., 2020; Wang et al., 2021b). BO generates a probability model of the objective function and uses it to determine the most promising hyperparameters to estimate the true objective function. BO is especially advantageous for problems where the true function is highly non-linear and difficult to evaluate its optimization. Therefore, BO treats the true objective function as a random function and applies a *prior* over it based on the existing data points. The *prior* is then updated based on the acquisition functions (AF) to form the *posterior* distribution over the true objective function. After that, the posterior distribution is used to construct a new AF and then determine the next query point. BO is accomplished by repeating the aforementioned steps until the maximum iterations to find the best surrogate model for the true objective function. AF used in BO is a trade-off between exploration (keep searching for new strategies) and exploitation (while exploring the best strategies found thus far), which is important to minimize the number of evaluated data points. Commonly used AFs are expected improvement, probability of improvement, and knowledge gradient (Chen et al., 2020). There are several methods used to define the prior/posterior distribution over the true function based on the sampled data points. The most common one is the Kriging method (Kleijnen and van Beers, 2020). BO is very well suited for functions that are expensive to evaluate either computationally or experimentally.

### 3.9 Pareto Active Learning

When targeting multi-task problems, most multi-task learning (MTL) approaches aim to find one single solution to optimize the overall performance of all tasks. However, it is observed in many applications that some tasks could be incompatible with each other, so no single optimal solution can optimize the overall performance concurrently. In real-world applications, MTL practitioners have to make a trade-off among different tasks (Alhammadi et al., 2004; Jablonka et al., 2021). Usually, no single optimum is preferred over all the others. Instead, there is a set of all Pareto-optimal solutions (or Pareto front) whose performances dominate the rest of the entire design set (Brisset et al., 2015). Therefore, Pareto active learning (PAL) is emerged as an active learning algorithm to find a set of Pareto optimality over every point of design space *E*. Moreover, a recently modified version of PAL called *ε*-PAL is able to predict the set of solutions that covers the true Pareto front of *E* with some granularity regulated by a parameter *ε* (Zuluaga et al., 2016). There are two main advantages of *ε*-PAL over traditional one. The value of *ε* allows us to generate an *ε*-accurate Pareto set, reduce the computational time by removing redundancy and rejecting points more efficiently.

## 4 Application of ML for Understanding and Design of Polymer Chains

In this section, we are going to discuss the most recent studies of ML applications in polymer chain characterization and inverse design by answering the following four key questions: *1*) *What is the bottleneck in polymer chain characterization or inverse design? 2*) *What is the ML strategy? 3*) *How can ML solve the challenging problem?* and *4*) *How can we leverage the model in future applications*?

### 4.1 Classification of Polymer Chain’s Configuration

In polymeric materials, some chain configurations are not distinguishable by direct visualization, especially disordered ones, yet there is a lack of clear local structural parameters or robust theoretical models for their classifications. ML methods have demonstrated their surprising capability in recognizing patterns of enormous complexity after being appropriately trained by humans or self-trained through learning mechanisms (Bishop, 2006; Schmidhuber, 2015). In this case study, a hybridization of ML and CGMD simulation was adopted to efficiently classify various configurations of polymer chains, including disordered, partially ordered, and ordered states (Wei et al., 2017). To complete this task, Wei et al. used a standard FNNs model in which the polymeric structures were used as inputs and corresponding labeled structural configurations were used as output. The output included gas-like coil, liquid-like globular, and crystalline *anti*-Mackay and Mackay structures. The CGMD simulations were performed to generate 5000 coarse-grained polymer configurations for the training process, and then a supervised ML algorithm was implemented to build a relationship between the input structures and their corresponding types of configuration.

**FIGURE 4:**
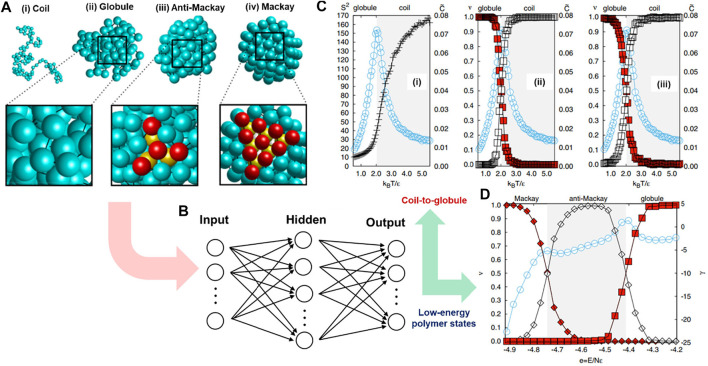
Identifying polymer states by machine learning. **(A)** Typical configurations of a polymer chain in states of **(i)** coil, **(ii)** globular, **(iii)** anti-Mackay, and **(iv)** Mackay. **(B)** Polymeric configuration is imported into a fully connected FNNs. The circles represent nodes connecting layer by layer via weight and activation parameters. The number of input nodes is 3N representing the numbers of spatial coordinates of the N-monomer chain, while the number of output nodes depends on the number of configurations that will be classified. **(C)** FNNs classifies polymer configurations under coil-to-globule transition: **(i)** Monte Carlo (MC) shows phase transition point from coil to globule at k_B_T/ϵ ≈ 2.0 corresponding to the peak of specific heat value C; the mean FNN output (ν values) can be correctly evaluated for globule (red squares) or coil (white squares) based on either original **(ii)** or normalized **(iii)** coordinates of monomers with the accurate transition points (k_B_T/ϵ) compared to CGMD simulations **(i)**. **(D)** FNNs model can classify Mackay (filled diamonds) and anti-Mackay (open diamonds) based on the mean FNNs output (ν values), correctly compared with transition points represented by a specific heat-like curve (Mackay-anti-Mackay-globule) derived from independent MC simulations. Figure was reproduced from Ref. (Wei et al., 2017) with permission.

Specifically, the input was the polymer’s 3D structure obtained from the CGMD simulation (Figure 4A). It was represented by 3*N* spatial coordinates of *N* bonded monomers (*N* = 102). In these MD simulations, the polymer model is a generic one. The bonded beads are connected by a particular implementation of the FENE model, and nonbonded monomers interact with each other via a LJ potential. The ML model (FNNs) contained three layers of nodes, including input, hidden, and output layers consisting of *N*_*i*_, *N*_*h*_, and *N*_*o*_ neurons, respectively. The 3*N* coordinates were fed directly into 3*N* input nodes of the *N*_*i*_ (Figure 4B). The FNNs model was trained to establish the relationship between the vector of monomer coordinates and the corresponding configuration in the output layer (*N*_*o*_). There were 100 nodes in the hidden layer (*N*_*h*_), and the number of output nodes was set to 2 or 3, depending on the number of polymer configuration types. By doing so, the model could classify both globule-to-coil (Figure 4C) and Mackay-to-*anti-*Mackay-to-globule (Figure 4D) transitions in a very convenient and robust way. It directly passed the unlabeled configuration (molecular coordinates) to neural networks without defining any order parameter or requiring high numerical precision methods (Wei et al., 2017). This hybridization of ML model and CGMD simulation in polymer configuration classification has opened numerous opportunities for similar research topics, including categorizing knot types of polymer conformation (Vandans et al., 2020), identifying the Gardner transition (temperature-induced transition) (Li et al., 2021), or even more complex polymeric systems such as entanglement effects or polymeric crystalization (Morthomas et al., 2017), and phase separation of block copolymers (Arora et al., 2021).

The CGMD/ML coupling can also learn and distinguish various global aggregate structures of sequence-defined copolymers. As mentioned in the Section 1, the monomer sequence governs the bulk self-assembly, which eventually influences the synthetic multiblock copolymer’s microstructures (Gody et al., 2016; Jiang et al., 2018). However, understanding the self-assembly behaviors of copolymer faces some issues once suitable order parameters are not well identified. As a result, ML methods are considered because they are well-known to be capable of building a rigorous structure-property relationship of materials (Liu et al., 2017; Ramprasad et al., 2017). However, due to the unknown numbers of disordered states of the copolymer, supervised learning is not appropriate, thus it requires alternative ML models to efficiently recognize random copolymer topologies. Thus, unsupervised ML algorithms are the most suitable ones for this classification task (Statt et al., 2021).

**FIGURE 5:**
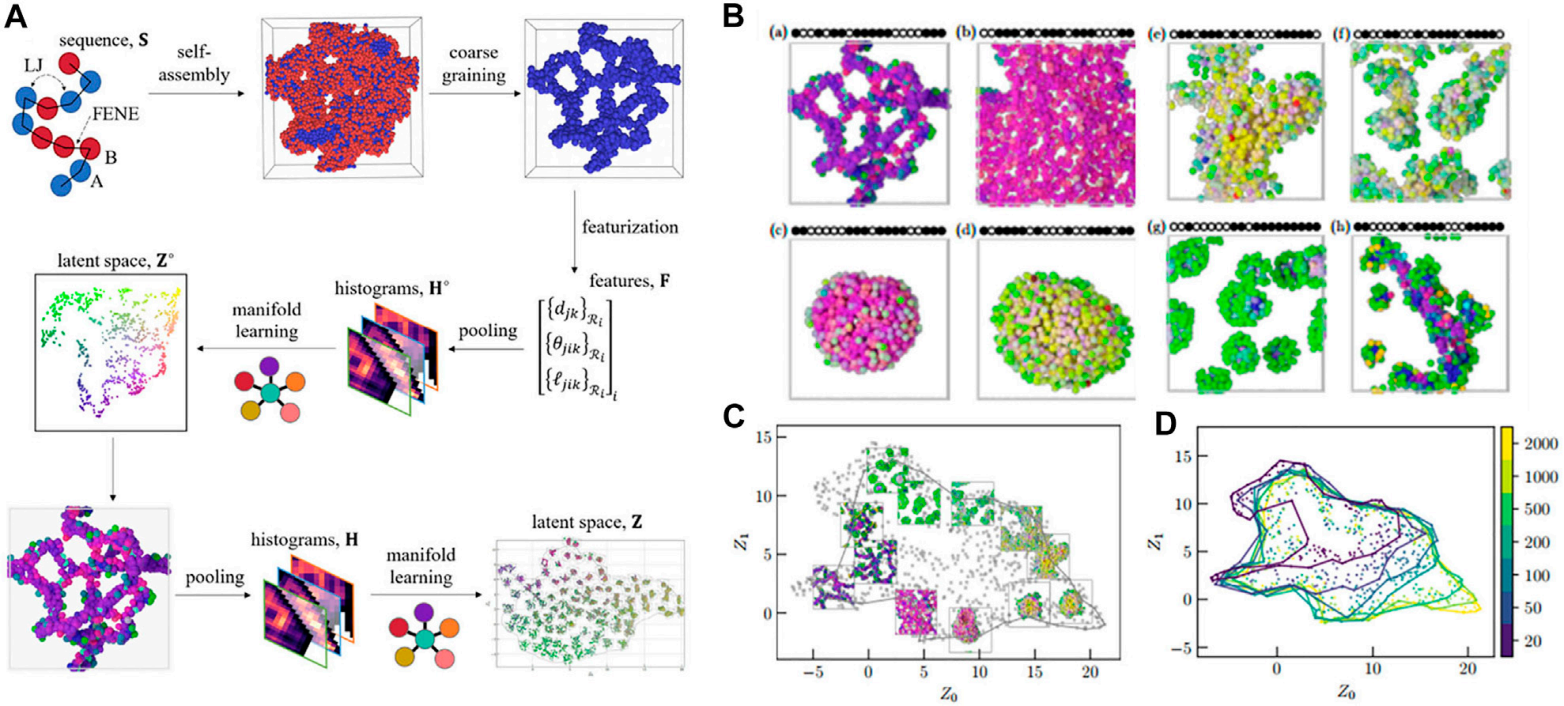
Unsupervised learning of aggregation behavior for a sequence-defined copolymer. **(A)** Schematic of unsupervised learning method for aggregation classification: the aggregation configuration containing 500 copolymer chains is used as an input, three-body features **F**_**i**_ is calculated for each bead which includes distance between neighbors, bond angle, and bond length (featurization) prior to carrying out Gaussian expansion and pooling over the features in order to obtain a histogram **H** ^**o**^. The histogram is then embedded into a latent space **Z** ^**o**^ to obtain local structures of the aggregation, before being implemented in a second pooling and projecting into a latent space **Z** in which all morphologies can be distinguished. **(B)** Representative structures perceived from the manifold learning including strings **(a)**, membranes **(b)**, vesicles **(c)**, liquids **(d)**, structured liquids **(e)**, disordered micelles **(f)**, spherical micelles **(g)**, and wormlike micelles **(h)**. Sequences are shown schematically, with • indicating an A-bead and ○ for a B-bead. **(C)** Representative configurations are embedded in the manifold with number of chains (system size) N = 500 showing a full range of possible structures for copolymer’s aggregation classification. **(D)** The alpha shapes for each system size (N = 20, 50, 100, 200, 500, 1,000, and 2,000) in the manifold reveal a gradually larger coverage in the latent space when increasing system size. Figure was reproduced from Ref. (Statt et al., 2021) with permission.

In principle, the ML methodology will employ feature vectors of copolymer configurations and embed these local descriptors into a low-dimensional manifold (latent space). The self-assembled structures will be afterward characterized and classified based on the feature vectors in the latent space. This methodology is called dimension reduction. In detail, CGMD simulation generated more than 2000 polymer aggregation configurations as inputs for the ML model (Figure 5A). One aggregation structure contained 500 chains of the copolymer. Each polymer chain consisted of 20 monomers of **A** (sticky) and **B** (non-sticky) with different bead types (Figure 5B). The MD simulation was implemented by a generic model. The sticky beads interacted with each other via the LJ potential, meanwhile the non-sticky interactions were described by the purely repulsive Weeks–Chandler–Anderson (WCA) potential. The bonded beads were represented by the FENE potential. For each monomer *i* in the polymer chain, local neighborhood ***R***_***i***_ was calculated using an isotropic cutoff radius with ***n***_***i***_ monomers inside the cutoff radius. From there, a structural input vector was calculated as three-body features ***F***_***i***_ between particles (*i*, *j*, *k*), including the distance between neighbors $d_{\mathrm{jk}}=\;\left|r_{k}-r_{j}\right|$ , bond angle $\theta_{\mathrm{jik}}=arccos\left(r_{ik}\cdot r_{ij}\right)$ , and bond length $l_{\mathrm{jik}}=d_{ij}+d_{ik}$ where ***r***_***ij***_ is the displacement vector between particle *i* and *j* for the entire neighborhood. All these feature vectors are translational and rotational invariant. Besides, the permutation invariance is enforced by performing the Gaussian expansion and pooling to yield a histogram of features $H_{i}^{o}$ *.* This histogram was then reshaped and embedded into a low-dimensional latent space ***Z***_***0***_ using a Uniform Manifold Approximation and Projection (UMAP) approach, which is a non-linear, unsupervised method for dimension reduction (Xiang et al., 2021)*.* By doing so, a projection of $H_{i}^{o}\;$ was obtained in the latent space ***Z***_***0***_, and then local structural information of monomers was achieved based on their positions in this manifold*.* The local information was then pooled and embedded once again, following the same procedure to generate collective variables in order that all copolymer morphologies could be observed and classified in the second latent space ***Z*** (Figure 5A). By using this strategy, Statt et al. showcased the ability to use the CGMD/ML hybridization to characterize global structure in disordered aggregation states based on local environment information, without explicitly considering global geometry (Figures 5C,D). Furthermore, this methodology is applicable in other soft materials, especially for understanding macroscopic self-assembly behaviors when suitable order parameters are not well defined, for instance, copolymer, peptide, and peptide-like systems.

### 4.2 ML Prediction of Polymer Property

**FIGURE 6:**
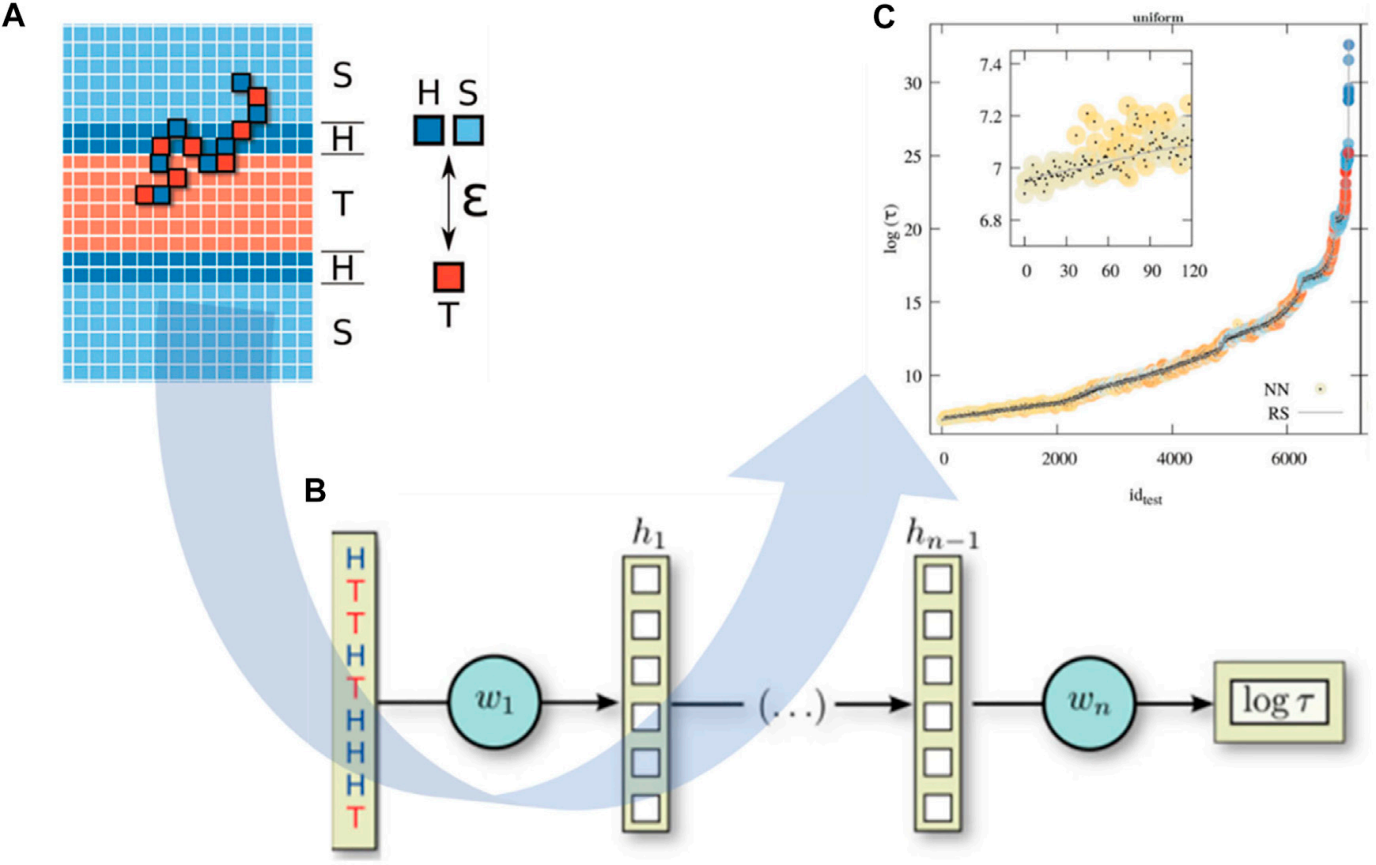
Neural network model for predicting copolymer translocation through amphiphilic barriers. **(A)** CG polymer chains are adopted in membrane translocation simulations with a laterally homogeneous membrane surrounded by solvent grids. Herein, H represents a hydrophilic grid, T for hydrophobic core, and S as solvent. **(B)** NNs model is used for predicting the translocation time of a polymer as a function of hydrophilic/hydrophobic (H/T) sequence. **(C)** NNs can predict accurately the translocation time of sequence-defined copolymer (dots) compared to RS-based results (gray line) with a root mean squared relative deviation in the order of 1% for the logarithmic mean translocation time. Figure was reproduced from Ref. (Werner et al., 2020) with permission.

The integration of CGMD/ML is able to speed up the prediction of polymer properties at the chain level. Researchers have adopted ML models for the prediction of polymer properties mostly based on their monomer representation (Ramprasad and Kim, 2019; Sattari et al., 2021; Chen et al., 2021b; Gracheva et al., 2021), ignoring the influence of polymer chains, such as molecular weight, topology (Tao et al., 2021a), and copolymer sequence (Kuenneth et al., 2021). Particularly for novel polymeric materials, there are limitations in the existing database due to unexplored chemical space (Wilbraham et al., 2019). Under these circumstances, CGMD simulation is very beneficial to generate new training data, which can be used for ML studies. One of the most recent studies in polymer property prediction is to estimate the translocation time of a copolymer through a lipid membrane as a function of its sequence of hydrophilic and hydrophobic units (Werner et al., 2020). Sequence-defined polymers have a wide range of applications in biomedicine and biotechnology for drug or ligand designs (Hartmann and Börner, 2009; Hartmann, 2011; Deacy et al., 2021). However, their translocation through lipid membranes and biological barriers has not clearly been studied with an accurate theoretical relationship between monomer sequences and their membrane-translocation ability. To overcome this challenge, Werner et al. used a DNNs model to unravel this structure-property correlation. In detail, more than 8,000 monomer sequences obtained from coarse-grained (CG) modeling (Figure 6A) were used as input to predict the corresponding translocation time as output (Figure 6C). The polymer simulation was a generic model where the monomers were represented as simple cubic lattices, and the CG polymer structure was placed in an external concentration field that represents a mean-field level bilayer membrane composed of an hydrophilic region (H) and a hydrophobic core (T), as well as solvent (S) (Figure 6A). Bond vectors were taken from a set of 26 vectors with lengths of 1, $\sqrt{2}$ and $\sqrt{3}$ lattice units. Double occupancy of lattice sites was forbidden, and the monomers had excluded volume. Additionally, short-range repulsive interactions were implemented on between hydrophilic sites (H and S), and hydrophobic sites (T). The DNNs model employed four consecutive hidden layers with the number of nodes as 64: 64: 32: 32 (Figure 6B). In the input layer, monomer sequence information was used as a vector of values 0 and 1 representing the sequence of hydrophobic and hydrophilic monomers, respectively. There was one node in the output layer representing the corresponding translocation time of the polymer. It was calculated based on the Rosenbluth-Rosenbluth (RS) sampling method for CG polymers through an external concentration field that represents a bilayer membrane structure (Rosenbluth and Rosenbluth, 1955). The DNNs model thereby accurately established a complex connection between the hydrophobicity and sequence-dependent translocational ability of a copolymer at the bilayer–solvent interface. Even though this work was focused on a simple CG polymer—membrane system, the model of DNNs is expected to enhance our predictive capability in a wide range of applications, for example, in complex biological systems such as nanoparticle–bilayer interactions (Wang et al., 2017), in characterizations of critical properties of polymer materials such as glass transition temperature, the radius of gyration, structural factor, stress-strain relation, etc.

**FIGURE 7:**
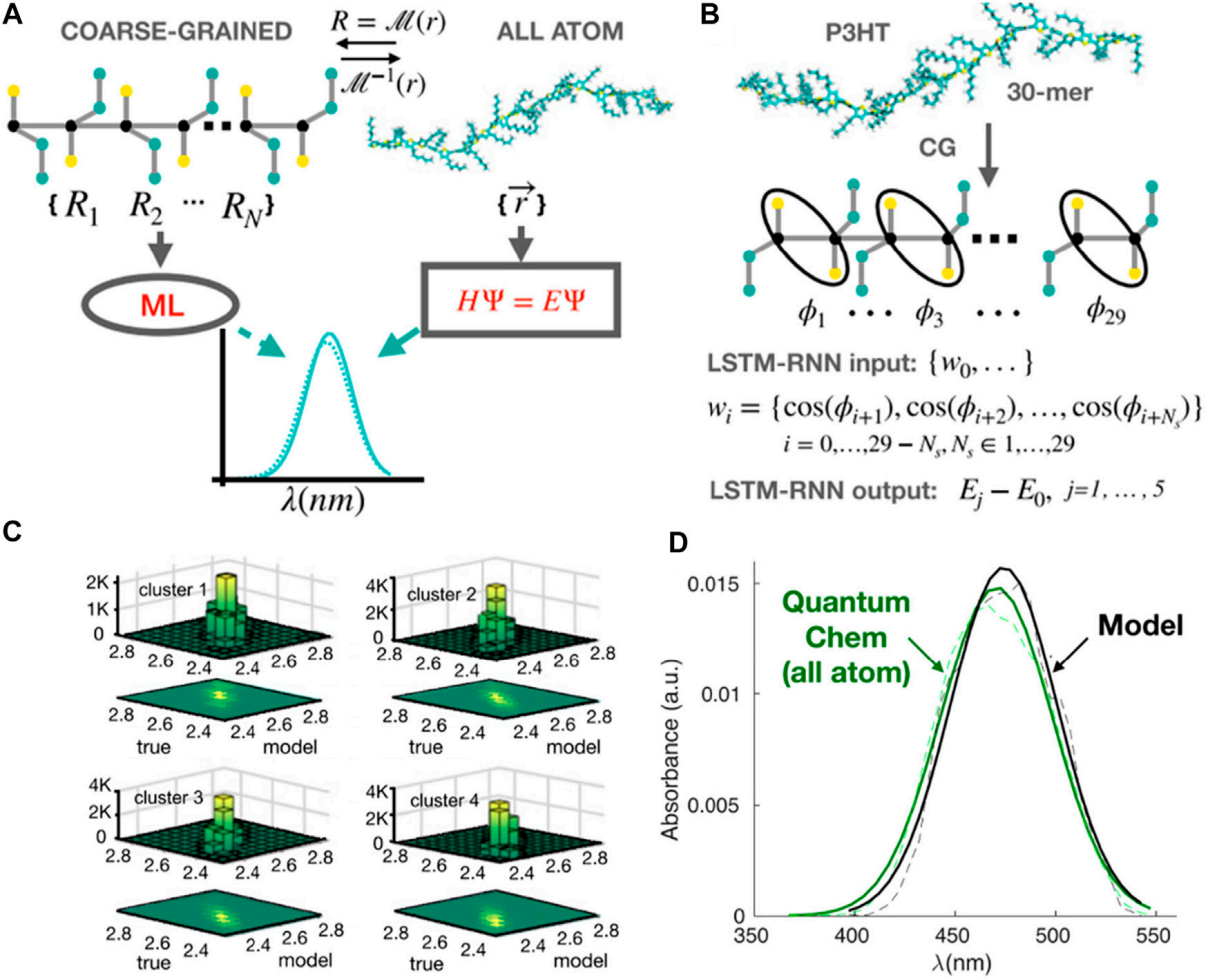
Predicting optical spectra for polymers using CG models coupling RNNs. **(A)** Scheme of ML method to predict spectra from CG representations of conjugated polymers, compared to the traditional approach using all-atom quantum calculation. **(B)** CG representations are back-mapped in order to obtain inter-monomer dihedral (φ) information. The sequence of cos φ along the backbone of the polymer is used as input of an LSTM-RNN model to predict the values of excited-state energies, then compared to all-atom quantum calculations. **(C)** Predictions of energies for the ground-to-first excited S_1_ state transition (E_01_) are validated in four distinct clusters (four distinct torsional subsets in the dataset) with the Pearson coefficient is ∼0.94 for all clusters (i.e., highly accurate prediction). **(D)** A comparison of spectra obtained using quantum chemistry from atomistic representations (green) and the LSTM-RNN model using inter-monomer dihedrals information (black): dashed line for raw statistical data and solid line after using Gaussian fits. Figure was reproduced from Ref. (Simine et al., 2020) with permission.

The CGMD/ML coupling can also predict optical properties of conjugated polymers, for example, UV-vis (light absorption) spectrum, one of the key experimental methods for characterization of conjugated polymers (Ivan et al., 2012; Root et al., 2017; Abdel‐Aziz et al., 2021). However, there is no well-established bridge between these CG polymer structures and their absorption spectroscopy. Therefore, using CGMD coupled ML models can answer the question of whether we can directly predict the UV-vis spectra of conjugated polymers from their CG representations (Simine et al., 2020). Since the polymer is a monomer-sequence of information, it leads to another question of which ML algorithm is efficiently used when it comes to sequential data? One of the most commonly used methods in natural language processing is LSTM-RNN (Hochreiter and Schmidhuber, 1997; Sutskever et al., 2014)*.* In particular, the monomeric information used in this study is dihedral twisting angles that are well understood to qualitatively define the electronic states and quantum energy of polymer structures. Hence, Simine et al. took advantage of this sequence of dihedral angles as molecular sequence information to represent the spectral energy of the whole polymer chain (Figure 7A).

In the LSTM-RNN model, the training and validation datasets were produced using CGMD simulations of 50 chains of poly-3-hexyl (P3HT) with 30 monomers. The force field governing the MD simulation model for polymers consists of harmonic bond, angle, and dihedral terms for the bonded atom interactions, as well as LJ interatomic potentials describing non-bonded contributions. Since the model was a chemistry-specific approach, CG parameters were tuned from AA model to reproduce the aggregation and optoelectronic behaviors of this polymer. The input was the torsional conformation of each CGMD configuration of the polymer (Figure 7B). It is a vector of 29 cosines of the inter-monomer dihedral cos (φ) taken from the back-mapped atomistic molecular structures of polymer. The output was the associated values of the ground-to-excited state *S*_*j*_ transition energy (*E*_*jo*_). These values were calculated using the all-atom quantum chemistry method called Pariser–Parr–Pople (PPP) model Hamiltonian (Simine and Rossky, 2017). The LSTM-RNN model employed one hidden layer with 150 nodes to establish a relationship between the sequence of torsional angle information and the corresponding energy-state deviations. This CGMD/ML coupling can effectively predict optical properties or UV-vis spectra for different conjugated polymers with high accuracy based on conformational information solely (Figures 7C,D). In the future, the model can be further applied not only for UV-vis or other structure-dependent spectral properties such as fluorescence, Raman, Infrared, etc. but for characterization of the bulk electronic and optical properties of photoactive materials.

### 4.3 Inverse Design of Sequence-Defined Polymers

Another application that has recently received significant interest is called polymeric inverse design or target design. However, like other soft materials, polymer design faces a major impediment due to the chemical, topological, and morphological complexity of macromolecular systems (Ferguson, 2018; Jackson et al., 2019; Sherman et al., 2020; Wu et al., 2020), as well as the proper representation or description of soft materials at macromolecular scale to make the calculations feasible (Audus and de Pablo, 2017; Peerless et al., 2018; Lin et al., 2019). Especially, in terms of sequence-defined copolymer inverse designs, the number of possible candidates goes up exponentially with the increase of chain length, requiring more effective tools for inverse design at the chain level. Recently, Webb et al. (2020) overcame this limitation by introducing a directed design of a copolymeric structure with branches and tailored sequences of monomers represented by CG beads. Sequentially, ML tools generated a surrogate prediction and target design for polymers with specific configurations based on the CGMD training dataset.

**FIGURE 8:**
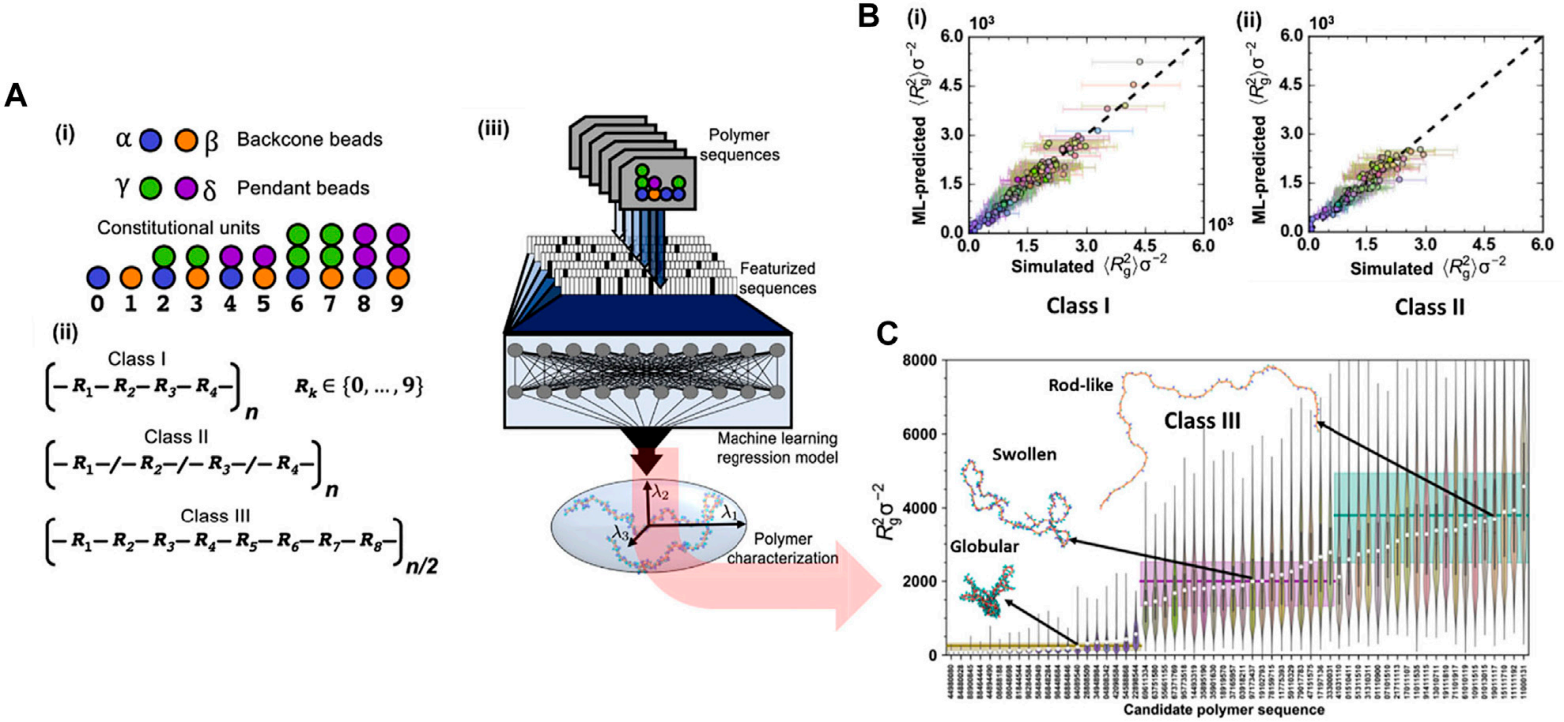
Inverse design for target polymeric configurations. **(A)** CGMD/ML coupling is used for the prediction of copolymeric radius of gyration (R_g_ value): **(i)** CG bead types used in MD simulation include backbone types (α or β) and pendant types (γ or δ). The allowable combinations of backbone and pendant beads yield 10 unique constitutional units (CUs); **(ii)** three classes of polymers are studied including class I, II, and III represented by different sequences of CUs: class I represents regular copolymers with a repeat pattern of four CUs, class II includes random polymers constructed from four CUs and class III constructed with a repeat pattern of eight CUs; **(iii)** Scheme of ML model for predicting polymer properties or R_g_ values. The entire polymer sequence or a repeat unit of it is featurized and used as input to a DNN model, which establishes the structure-property relationship of the polymer. **(B)** Performance of ML model for prediction of the mean square radius of gyration, 〈Rg^2^〉, for class I and class II polymers: **(i)** the prediction for class I is validated by CGMD modeling with the *r* ^2^ of 0.953, and the mean absolute error (MAE) of 111.32; **(ii)** the model trained on class I is applied for class II prediction with the *r* ^2^ of 0.895, and the MAE of 130.34. **(C)** Targeted sequence design of class III polymers using the Tree-structured Parzen Estimator (TPE) algorithm. The result presents three sets of recognizable predictions, with the globular polymers (first 20 polymers from the left) separated from the swollen targets (next 20 targets), which are easily distinguishable from the rod-like structures (last 20 targets) as well as fit with the statistical distribution of R_g_^2^ obtained from CGMD validations (in the form a violin plot). Figure was reproduced from Ref. (Webb et al., 2020) with permission.

This study aimed to achieve two most important goals: prediction and inverse design of polymer chains. For the former task, a DNN model took features from more than 1000 CG **class I** polymeric sequences as input to predict the value of the radius of gyration (*R*_*g*_) for both classes I and II polymers. For the latter one, the Tree-structured Parzen Estimator (TPE) algorithm (a Bayesian Optimization approach) was used to generate novel candidate sequences of class III polymer for target values of *R*_*g*_ based on sequential model-based optimization technique (SMBO-TPE) (Bergstra et al., 2011). The MD simulation model for polymer was a generic one in which the polymer interactions were described by summation of typical bonded and nonbonded potentials. The bonded interactions include stretching energy (FENE), harmonic angle bending and torsinal energies. In the DNN model, the featurization input could be simple one-hot encoding (OHE) or property coloring. In the OHE approach, each constitutional unit (CU) was represented as a 10-bit vector (10 different constitutional units or CUs) with a single high element corresponding to a specific CU type (Figure 8A). In all cases, featurized input was based on either a repeating subunit of the polymer or the entire polymer sequence. For example, **class I** polymers could be defined using a constitutional repeating unit of four CUs, which is represented as a 40-bit OHE vector. However, for stochastic sequences of polymers (**class II**), featurizing the entire sequence was required. In the colored-property approach, the featurization is more flexible where the entire polymer chain was encoded as an image, with each bead of polymer represented by a pixel with coloring determined by local properties. The image was afterward used as input for a CNN model to generate featurized input vectors for particular polymer sequences. All regression models used two hidden, fully connected layers with 20 nodes followed by a single output node for the *R*_*g*_ predictions. By doing so, the ML model could accurately predict the *R*_*g*_ values for both **classes I** and **class II** polymers (Figure 8B). For the target design on **class III** polymers based on *R*_*g*_, the TPE algorithm generated a candidate sequence, compared the estimated mean square value of the radius of gyration 〈Rg〉 from the ML model to the target, and then proposed a new sequence based on historical performance. The targets included globular, swollen, and rod-like polymeric structures. For each target, 20 candidate sequences of the **class III** polymer type were created, and their radius of gyration *R*_*g*_ values was subsequently validated using CGMD simulations (Figure 8C)*.* By combining CG modeling, ML, and model optimization, the methodology could certainly predict structural properties with limited sequence information and further successfully design the targeted polymer sequences (globular, swollen, or rod-like behaviors). This powerful integration addressed the challenges related to soft material inverse design, where chemical and topological information is broad and puzzling to be computationally manageable. This work also highlighted its significant potential for designs of novel polymer-based materials or sequence-specific systems in a tailored region of the polymer genome (Kim et al., 2018).

Peptide design is somehow similar to sequence-defined polymer inverse design in which the targeted properties depend on the arrangement of amino acids. For example, the self-assembling behavior of π-conjugated peptides influences its optical and electronic properties in biological environments (Kim and Parquette, 2012; Guo et al., 2013; Pinotsi et al., 2016). The self-assembling activity of these peptides is particularly governed by tunning its molecular chemistry of the π-core and the sequence of amino acids of the wings (Mansbach and Ferguson, 2017). However, this task still faces challenges because the sequence-structure-function relation of the peptide remains poorly identified, due to the great extent of the number of possible sequences for evaluation (Shmilovich et al., 2020). In this work, Shmilovich et al. considered a design of a peptide family of *DXXX-OPV3-XXXD* in which there were 8,000 possible sequences. The biggest question that arose was how to achieve a targeted design from such a vast size of chemical space efficiently? Trial-and-improvement experimentation is essentially intractable due to the significant time and labor costs associated with peptide synthesis and testing. On the other hand, brute-force simulation of all possible structures is unfeasible, even though CGMD is known to be advantageous for macromolecular characterization. Therefore, the coupling of CGMD with ML technique can tackle that issue by only focusing on the most promising candidates within the peptide family.

**FIGURE 9:**
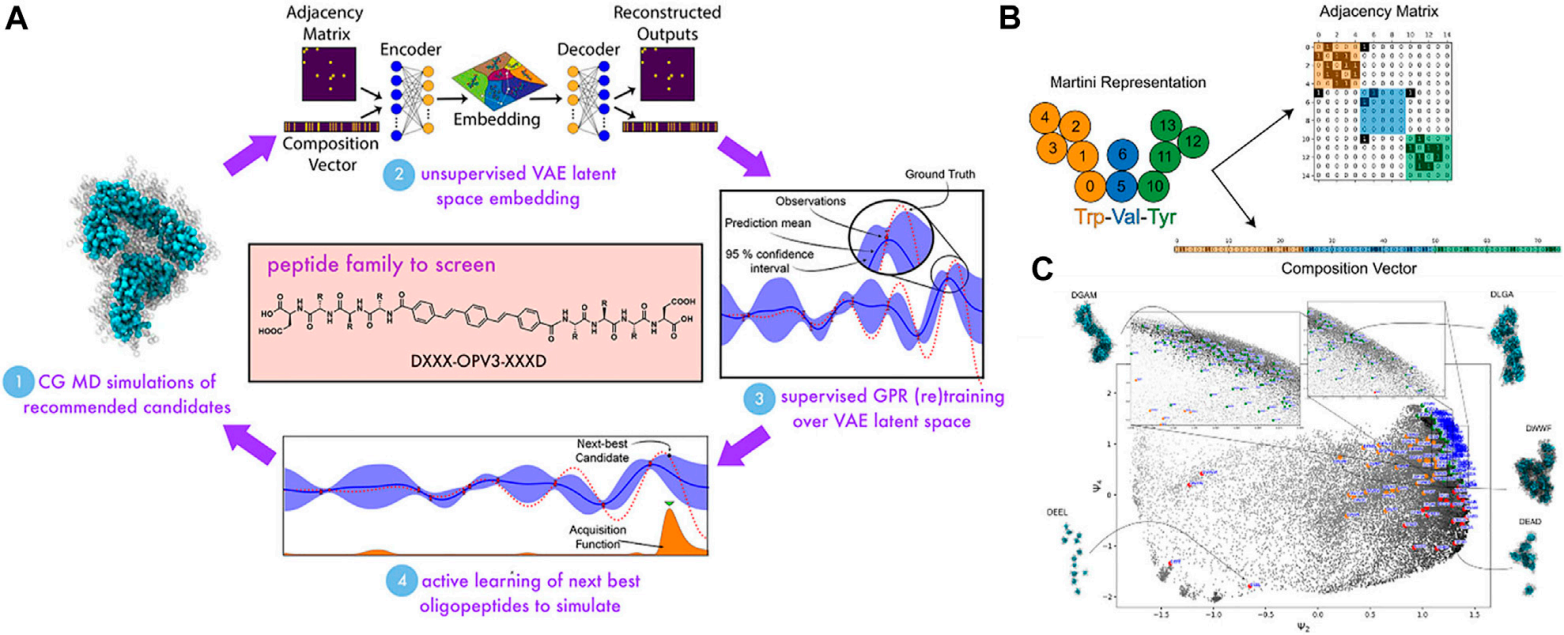
Target design of self-assembling π-conjugated peptides using active learning algorithm. **(A)** Scheme of active learning approach (Bayesian optimization) in search of DXXX-OPV3-XXXD peptides with optimally self-assembling behaviors: **(1)** CGMD simulations are performed on 90 randomly selected candidates of the family and their self-assembling abilities are measured based on fitness function f; **(2)** The self-assembled aggregates from high-dimensional chemical space are projected into a low-dimensionality manifold (latent space) using a variational autoencoder (VAE). The dimensionality of the latent space is optimized after each cycle via minimizing the VAE loss function; **(3)** A Gaussian process regression (GPR) model establishes a relationship between the latent space coordinates of peptides and their fitness function f which indicates the self-assembled property of peptides. The GPR model improves every cycle (with an additional training point); **(4)** An acquisition function is used to predict the “next-best” DXXX-OPV3-XXXD candidates for which to run CGMD simulations to direct sampling toward the most promising candidates. The active learning loop is cycled until the GPR surrogate model has no more updates with the additional training data points. Eventually, the top candidates predicted from the active learning will be validated using explicit MD simulations. **(B)** The representation of each XXX tripeptide (e.g., Trp-Val-Tyr) to the VAE includes an adjacency matrix **A**_**i**_ indicating the connectivity of Martini beads within the tripeptide and a one-hot encoded composition vector **T**_**i**_ representing the identity of the beads. The couple **(** **A**_**i**_, **T**_**i**_ **)** is then used as the input of the VAE embedding. **(C)** Snapshots are harvested from all molecular simulations (558,000 in total) projected into the ψ_2_-ψ_4_ plane (top three nontrivial diffusion map eigenvectors). The result showcases natural categorization for self-assembling behaviors represented by colored points (green for good assemblers, red for intermediate assemblers, and orange for poor assemblers), which demonstrates design rules to promote good assembly behaviors. Figure was reproduced from Ref. (Shmilovich et al., 2020) with permission.

Among ML techniques, Bayesian optimization is one of the most common active learning method. It is able to steer the experiments or simulations toward “next-best” candidates based on historical measurements (Chen et al., 2008; Ling et al., 2017; Gómez-Bombarelli et al., 2018; Barrett and White, 2021). The first step is to define a fitness function that evaluates a particular property. In this study, in order to evaluate the self-assembled aggregate capacity, a metric called “*optical distance*” was used, defined as the minimum center of mass distance between aromatic cores of every two molecules in a peptide aggregation (total of 96 peptide chains) obtained from CGMD simulations. In this chemistry-defined MD simulation, the popular Martini potential was used to described the interactions, including bonded and nonbonded between CG beads. The ML technique was supposed to find the best candidate with maximized fitness function or optical distance that promotes the peptide optoelectronic functions (Figure 9A). The next step was to use variational autoencoders (VAE) in order to convert the original peptide configurations into a latent space to make the optimization more robust and efficient (Gómez-Bombarelli et al., 2018). The peptide was represented based on XXX CG bead and specified using adjacency matrix ***A***_***i***_ which indicates the connectivity of beads and a one-hot encoded vector ***T***_***i***_ which demonstrates the composition of the CG beads in the peptide (Figure 9B). The couple of (***A***_***i***_, ***T***_***i***_) was used as input for the VAE which includes two parallel networks to extract input features. The decoding part then strove to reconstruct the (***A***_***i***_, ***T***_***i***_) from the latent coordinate ***z***_***i***_ using two parallel networks. The VAE would be trained to minimize the VAE loss, including a reconstruction term and a Kullback−Leibler divergence term (Prokhorov et al., 2019). After that, a Gaussian process regression (GPR) surrogate model was used to predict the fitness function ***f***_***i***_ of all unsimulated sequences depending on their local positions in the VAE latent space. A Gaussian process was employed to define a Bayesian prior distribution over the regression functions fitting the existing data points. The posterior distribution over those functions was updated as additional training data were sampled. After that, the next sampling was guided toward the next-best candidates based on an acquisition function embracing the current surrogate model to identify peptides with a high chance of being better than the current dominator in the training data. Here, the expected improvement function (EI) was used to provide a trade-off between exploitation (the area with the large posterior mean) and exploration (the area with the large posterior variance). The candidate with the highest value of EI would be selected next to perform CGMD simulation or some other expensive evaluations. The active learning was looped until that the GPR model was no longer better with the additional sampling. By doing so, the authors could identify top candidates that were predicted to exhibit dominating assembly by carrying out CGMD simulations for only 2.3% of the entire design space (Figure 9C). Their workflow reflected potential savings in time and labor afforded. This platform is promising for the design of other peptides, peptide-like, and sequence-defined polymer systems with optimized or desired properties, where only small numbers of top-performing candidates are identified.

In many real applications, scientists and engineers often encounter the challenge of how to optimize several independent objective functions simultaneously due to the fact that optimizing one objective alone can be incompatible with others (Clancy, 2020). To address this tricky question, they usually attempt to search for a set of materials where their performances on all of the objectives are superior to others in the entire design space. This material set is called Pareto-optimal solutions (or a Pareto front). To efficiently identify the Pareto front at low cost, i.e., by evaluating as few designs as possible, *ϵ*-Pareto active learning (*ϵ-*PAL) algorithm is exemplified to be competent for this task in which the usage of parameter *ϵ* allows us to control the accuracy of the prediction produced by the algorithm (Zuluaga et al., 2016). Recently, *ϵ-*PAL was implemented by Jablonka et al. to compute a set of Pareto optimal materials with multiple objectives and desired accuracy for a dispersant inverse design application (Jablonka et al., 2021).

**FIGURE 10:**
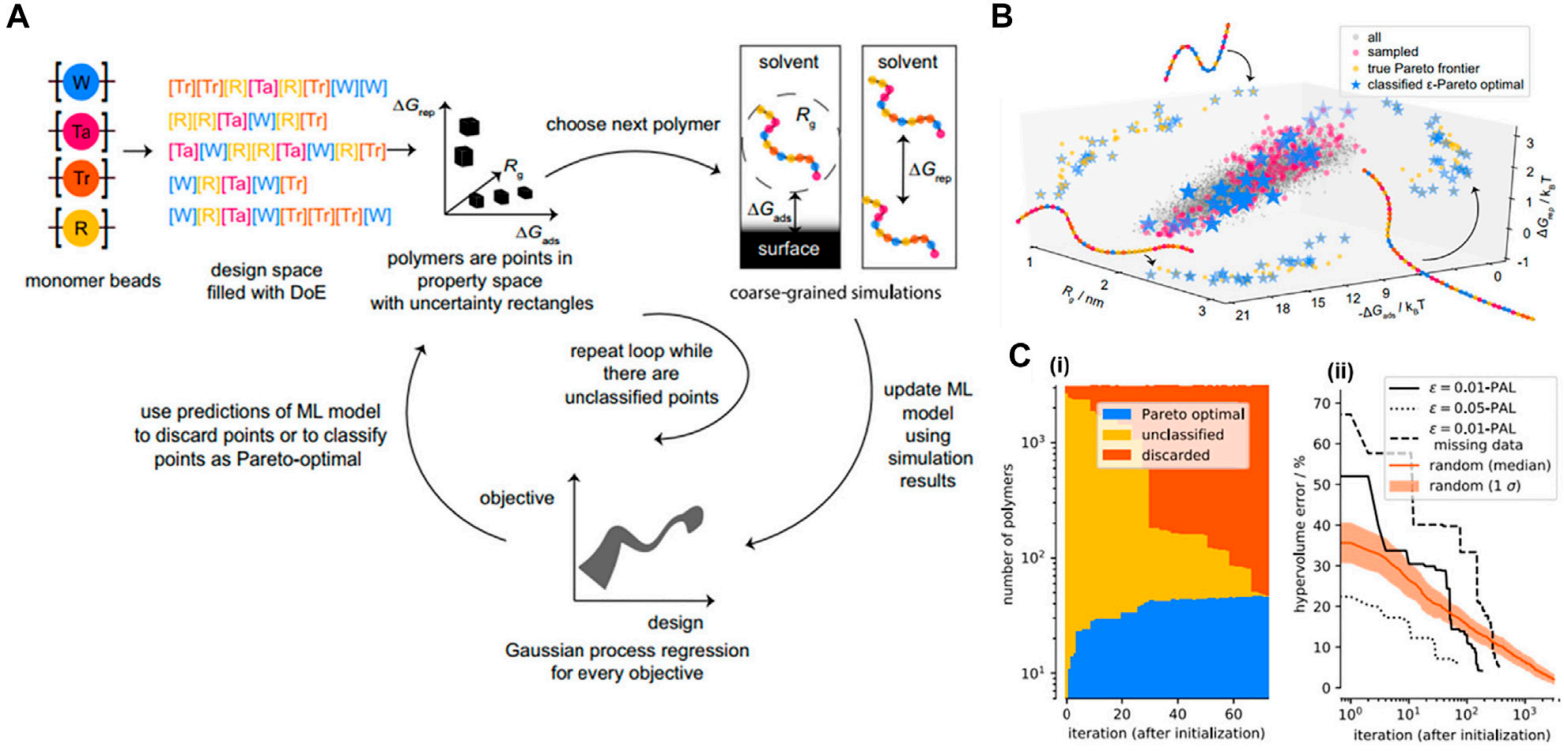
Active learning algorithm uses the Pareto dominance relation for dispersant inverse design applications. **(A)** Overview of the workflow. Representative samples are enumerated in the design space of sequence-defined polymers containing four different bead types: the “[W]” bead represents a polymer in a good solvent, the “[R]” bead to a polymer in a bad solvent, the “[Ta]” and “[Tr]” beads correspond to polymer in a theta solvent but differ from each other in their ability to interact with the surface. The entire design space consists of 3,125 polymers in total. For this ϵ-PAL algorithm, 60 initial samples are imported into a Gaussian process surrogate model to predict means and standard deviations that allow us to confidently discard points or classify them as Pareto optimal and especially decide which points should be performed on the CGMD simulations next to maximally reduce the uncertainty for the unclassified points near the Pareto front. The loop is cycled, and the Gaussian surrogate model is updated until there are no longer unclassified points in the design space. **(B)** Representation of polymers in property space: all data points in the entire experimental design space are determined with three key properties including the adsorption free energy (ΔG_ads_), the dimer free energy barrier (ΔG_rep_), and the radius of gyration (R_g_). The predicted Pareto front (blue) is well fit to the true Pareto front derived from brute-force simulations (yellow) demonstrating the effectiveness of this ϵ-PAL algorithm. **(C)** Classified points and hypervolume error as a function of the number of iterations. **(i)** illustrates the working principle and effectiveness of the algorithm: this method is able to classify the polymer as a Pareto optimal or a discarded point as fast as possible. After only ten iterations, the model can confidently discard a large number of polymer (orange) as well as effectively find many ϵ-accurate Pareto-optimal points (blue) in the design space. The number of unclassified points is reduced significantly at the same time (yellow); **(ii)** The performance of this algorithm is much faster (use much less iteration number) when compared with a random search using hypervolume error to quantify the convergence of Pareto front classifying. Especially when using a single surrogate Gaussian process model (for multiple-objective prediction) the model is able to perform better (compared to using multiple surrogate Gaussian process model) in case one of the objectives is missing while all other properties are present for a given data point. Figure was reproduced from Ref. (Jablonka et al., 2021) with permission.

The set of Pareto front in multi-objective dispersant design was supposed to dominate the others in three key properties obtained from CGMD simulations: 1) single-molecule free energy of adsorption onto a model surface (Δ*G*_ads_), 2) dimer repulsion energy (Δ*G*_rep_), and 3) radius of gyration which is an indicator of polymer viscosity (R_g_). The MD simulation model for polymer was a generic approach in which the interactions between monomer beads were described using a DPD approach including soft repulsive force, dissipative force, random force and an additional spring force term. In principle, the Pareto classification was expected to predict a Pareto set from a total of 3125 CG linear polymer structures (full design of space) based on the uncertainty estimate (σ) derived from a GPR surrogate model. As shown in Figure 10A, the model was started with a set of diverse experiments with measured objectives (experimentally or computationally), and then an initial model for every objective would be trained using a GPR model (a design—objective surrogate model). All the polymer points would be placed in a multi-objective space. For each point, the hyperrectangles were constructed from the surrogate model with a width proportional to the uncertainty *σ* corresponding to the points, i.e., an unsampled point would have a larger hyperrectangle than a sampled one. The points were then identified as those that could be discarded with confidence and those of which were with high-probability Pareto optimal based on a Pareto classification criterion (Jablonka et al., 2021). The loop was repeated until there were no unclassified points in the entire design space. The algorithm demonstrated its strong capability to identify the set of optimal points quickly in a multi-objective space with confidence and time-and-cost efficiency (∼89% fewer iterations) compared to the random exploration method (Figures 10B,C). This work can help significantly accelerate the process of exploring or optimizing materials for multi-task designs. The vision behind this approach is the applications for multiple-objective drug and polymer designs in the future, while simultaneously giving us insights into structure-property relationships and being robust under the circumstances of missing data or very expensive evaluations.

**FIGURE 11:**
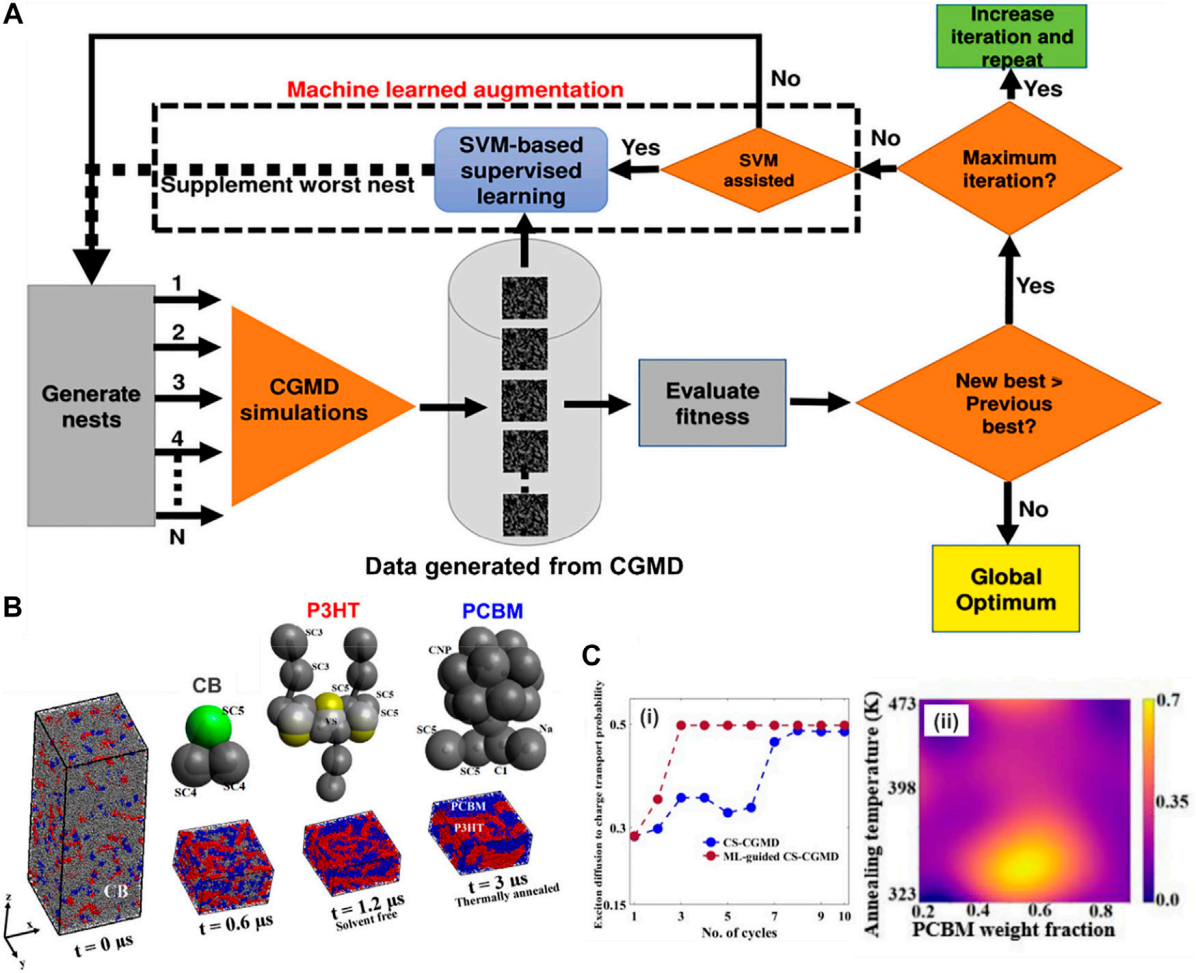
A coupling of ML and CGMD for a design of an organic photovoltaics (OPV) device. **(A)** Flowchart showcases the coupled ML—CGMD algorithm for OPV design. During each optimization generation, CGMD/ML compares different solutions among different nests and keeps the best candidates. Bad solutions are replaced with newer ones in the design space. The ML augmentation guides the design to the regions of interest (ROI) to replace the worst nests with the better candidates during each optimization cycle. The training dataset is also updated with new CGMD data after each optimization run. The support vector machine (SVM) works as a steerer for the CGMD simulation towards to best candidates. It plays a similar role to the Bayesian optimization as discussed earlier. **(B)** CGMD representation of phase separation of the mixture of poly-3-hexyl-thiophene (P3HT) (red) and phenyl-C61-butyric acid methyl ester (PCBM) (blue) solvated in chlorobenzene (CB) (gray) from the initial state to partial evaporation of the solvent (t = 0.6 µs), solvent-free (t = 1.2 µs) and thermally annealed (t = 3 µs). CB, P3HT, and PCBM molecules are presented following Martini force field coarse-grained beads. **(C)** Performance of the integration of ML and CGMD for OPV application. **(i)** A bivariate optimization of PCBM weight fraction and annealing temperature for improved exciton transport probability. The SVM assisted—CGMD converged much faster than the traditional approach after only 3 cycles; **(ii)** Landscape of the Pareto solutions from ML-CGMD integration for multi-objective OPV design: improved charge transport probability and increased ultimate tensile strength which are evaluated using CGMD simulations. Figure was reproduced from Ref. (Munshi et al., 2021) with permission.

The final case study in this review will help to answer the question of whether the integration of CGMD simulations and ML algorithms can be applied in real polymeric systems. Different from the aforementioned works which focused on the monomer sequence-specific inverse designs, this study investigated the influence of various compositions of polymers and other design parameters on the performance of an organic photovoltaic (OPV) device for solar energy conservation (Munshi et al., 2021). The OPV is considered an efficient alternative for solar energy materials (Scharber and Sariciftci, 2013). The device contains a phase-separated mixture of two organic molecules which accelerates with the exciton conversion and electron transport. However, the maximum of the OPV performance is at 15–20% and thus restraining its potential of solar energy (Balasubramanian et al., 2021). Therefore, solving an OPV design problem to make them more efficient is still a challenge, but also an attractive target for exploration. The biggest concern is about the existence of various design variables such as active layer thickness, the composition of polymers along multiple targeted properties, e.g., light absorption, charge diffusion and collection need to be optimized simultaneously. Even though, Balasubramanian et al. previously narrowed down the largest effects on the OPV’s overall efficiency to mostly two design variables including the annealing temperature and the proportion of the polymers in the design (Munshi et al., 2019), yet it is still an issue to efficiently optimize the OPV design with the fundamental trial-and-error approach. To tackle this bottleneck, Joydeep Munshi et al. coupled an ML searching algorithm with CGMD data generation which could help to robustly accelerate the design process for this solar energy device (Figure 11A). The authors attempted to optimize concurrently the compositions of the donor and acceptor polymer materials, and the annealing temperature for the highest power conversion efficiency (PCE). Particularly, the used material was a mixture of poly-(3-hexylthiophene) (P3HT) as an electron donor and phenyl-C61-butyric acid methyl ester (PCBM) as an electron acceptor (Figure 11B). Previous works have demonstrated a correlation between polydispersity index (PDI) of this polymer system resulted in various material performances (Munshi et al., 2019; Balasubramanian et al., 2021). Focus on finding the relationship between these key design variables (PDI and annealing temperature) that contributes to an improved PCE, this integration of ML and CGMD data generation allowed us to acquire a set of Pareto solutions for simultaneously enhanced charge transport probability and ultimate tensile strength of the material.

The input of the ML model was generated from the CGMD simulations. Initially, a mixture containing randomly P3HT and PCBM CG beads with different PCBM weight fractions was inserted in a simulation box of 20 × 20 × 80 nm^3^. The solvent evaporation and thermal annealing modeling were subsequently performed to obtain the phase-separated polymers with bulk-heterojunction (BHJ) morphology with a total simulation time of ∼3 µs (Figure 11B). Since this MD simulation was also a chemistry-specific approach, the Martini force field was adopted to model the intermolecular interactions between beads in the polymer system. The CGMD morphology evaluations were then performed by calculating the exciton diffusion to charge transport probability (CTP) and the ultimate tensile strength (UTS) under an applied deformation. The details of these calculations are given in the main manuscript and the supplemental information of Ref. (Munshi et al., 2021). The values of CTP and UTS were used as the objective functions for this CGMD/ML scheme. The output of the model was the global solution with optimized single- or multiple-objective (Pareto solutions) design which utilizes the BHJ morphology prediction from CGMD modeling. The cuckoo search (CS) optimization (Yang and Suash, 2009) with an ML-guided regression approach was used to steer the selections of promising eggs (solutions) during each optimization generation. The CS algorithm generated different nests per cycle in which the design variables (such as polymer mass fraction) were varied prior to implementing the CGMD simulations on these variables. A support vector machine (SVM) with radial basis function (RBF) fitting was used to pick the best candidate based on the CTP and UTS evaluations among CGMD simulation morphologies from all the nests. The best solution was next used to replace one of the worst-performing nests from the previous generation. By looping the process, the coupling of ML and CGMD could generate and compare different solutions amongst the different nests and retain a set of the best candidates. Ultimately, all the poor-performing solutions were replaced with better ones in the design space. Compared to the traditional searching method (without using SVM for guiding the solution selection), this integration could converge much faster (Figures 11C (i)) to identify the optimal conditions of the annealing temperature and PCBM weight fraction for maximizing a single objective (such as CTP or UTS). More interestingly, this methodology established a set of Pareto solutions in which multiple objectives (CTP and UTS) were superior at the same time in a range of optimal conditions of the annealing temperature and the polymer mass fraction (Figures 11C–11C (ii)). This work highlights the capability of the CGMD/ML toward more practical polymer blend design, such as solar energy conservation, battery electrodes, nanocomposite materials, etc. where there are numerous design parameters as well as multiple objectives to be taken into account. This methodology may provide better guidance for experimentalists compared to conventional approaches with significantly reduced cost and time.

## 5 Challenges and Future Directions

Although the applications of ML algorithms and CGMD simulations for polymer chains have been advanced recently, many questions remain to be addressed. From our perspective, four main topics as follows are considered as the most challenging:

### 5.1 Molecular Featurization

Homopolymers are usually characterized using a single repeating unit, and there are standard featurization methods such as substructure fingerprints (Morgan fingerprint) and physiochemical descriptors (Tao et al., 2021b). However, when converting copolymers that have multiple components into numerical vectors for ML models, it is not straightforward in terms of how to integrate the contributions of all components properly, particularly considering their sequence on a polymer chain. If based on each constitutional unit’s featurization like Morgan fingerprint, the most straightforward way is to use the weighted summation of their featurization vectors based on their composition proportions in the copolymer (Pilania et al., 2019). This strategy leads to a total vector that is invariant to the arrangement of monomer components in copolymers, which is only applicable to random polymers where no sequence order is involved (Kuenneth et al., 2021). For maintaining such feature invariance for different component permutations in random copolymers, the weighted summation method can be replaced by DNN networks to do mixing and aggregating, and the standard fingerprint can be changed to embedding networks to do feature representation learning (Hanaoka, 2020). For more applications where CG bead sequence affects the properties of copolymers (sequence-defined copolymers), explicit-sequence featurization strategies are preferred. One solution is to use an adjacency matrix that is able to represent the polymer connectivity and sequence, in the context of a graph representation of copolymers. Another solution is to arrange the feature vector of each component in order to form a larger vector, then a CNN model’s sliding kernel is able to extract sequence-level features (Patel et al., 2021). For CGMD/ML coupling, it is crucial to employ a proper molecular featurization that is able to consider the underlying chemical information of each component, the feature invariance for random copolymers, or the CG bead ordering for sequence-defined copolymers. Considering other types of copolymers such as gradient copolymer, block copolymer, or graft copolymer, whether weighted summation method or CNN model is still appropriate remains unknown. Incorporating CG bead ordering in the ML model is a fundamental yet not fully addressed challenge. More challenges lie in the combination of the multiscale complexity of copolymers and the topology complexity in the following.

### 5.2 Topology

Recent CGMD/ML coupling research focused mostly on the monodispersity with being limited to the short and linear polymer chains. However, experiments expect to see the polydispersity in polymeric topologies that would eventually affect the bulky self-assembly behaviors (Lynd and Hillmyer, 2005; Lynd et al., 2008; Vleugels et al., 2020). This polydispersity will be a potential design parameter to drive certain self-assembly pathways for copolymer structures and will need more investigations in the near future. A single chain with a limited number of monomers/particles has also been favorably used for the inverse design problem. It raises a related issue whether this CGMD/ML hybridization can be adapted for other complex polymeric systems? One suggestion is that when chemical complexity increases, more features are better to be incorporated, combining atomic connectivity, chain-level characterizations, degree of polymerization, morphological descriptors, etc. Another consideration is to differentiate CG backbone beads and pedant beads so that the linear and non-linear topologies are better recognized. Furthermore, more flexible ML approaches must be applied, such as using property coloring schemes representation for polymeric structures in two-dimensional (2D) convolution networks (Gao et al., 2018; Yang et al., 2018), or the use of graph convolutional networks (Coley et al., 2019; Korolev et al., 2020) as well as more powerful tools to handle various types of polymer chains, such as linear, branched, ring, star polymers. With the increasing number of features at a different scale, the CGMD/ML hybridization is expected to better reveal the structure-property relationship of a complex polymeric system, but it is not guaranteed. The performance of an ML algorithm itself is problem-dependent, not to mention the interplay between ML algorithm and CGMD configurations. Such complexity requires careful consideration of the system’s topology and consequently the features to be considered.

### 5.3 Model Accuracy and Transferability

Another tricky question is about the prediction accuracy of these models when being applied in unexplored corners of sequence space. It needs more investigation in the future on the relation between training data bias (data diversity) and the systematic prediction errors of models. For inverse designs, another challenge is whether the existing CG force fields are able to represent the polymeric structures accurately with enough chemical insights for real-life polymeric design applications (Li et al., 2013). This might require model parameterization as part of the design workflow to enhance the capabilities and accuracy of CG models to generate comprehensive training datasets for ML tools. On the other hand, back mapping of the CG model to the all-atom model might preserve information related to the molecular compositions of these polymers (Li et al., 2020a). One more related issue is how many CGMD data points will be sufficient for the ML training process? It has been known that active learning tools such as Bayesian optimization or Pareto searching algorithms can perform effectively on small datasets, yet it is only for inverse design. How to deal with that problem when it comes to the tasks of classification and feed-forward property prediction? Therefore, it requires more efforts to investigate the influence of the size of the training dataset on the model accuracy as well as setting up the criteria for the design of experiments (DOE) to acquire more effective samplings. Since the chemical space for copolymer is almost infinite, the evaluation of the model accuracy and transferability are always limited to the chemical space that is being investigated. Generating more data to cover a broader area of the chemical space leads to a brute force solution so that we can evaluate the model accuracy and transferability to the most extent. More importantly, it is better to generate a dataset as diverse as possible, with which the model accuracy and transferability become more convincing. Last but not least, we have known that CGMD still cannot fully represent the experimental observations quantitatively due to the challenges of length and time scales. Therefore, in order to predict accurately experimental phenomena, the algorithm and computing capability themselves need to be improved as well (Li et al., 2020b).

### 5.4 Combination With Experimental Results

Another concern is whether this methodology can be used in real-life manufacturing where processes take into account multiple parameters. In this situation, the existence of experimental data plays an important role. Recent research shows that integrating machine learning with experimental data allows us to accurately predict the areal proportion of each of the four morphologies in block-copolymer phase separation, identify critical process parameters, and predict the experimental outcomes (Tu et al., 2020). The experimental data is considered as a small set of high-fidelity data (Chen et al., 2021c), while the CGMD simulation provides a larger set of lower-fidelity data. Therefore, the multi-fidelity combination model is expected to enhance the performance of ML tools in reality (Meng and Karniadakis, 2020). Additionally, experimental data can also help to parameterize the CG potentials for a more consistent and reliable CGMD/ML methodology (Simine et al., 2020). It is worth noting that special attention needs to be paid to the experimental uncertainty. If experimental measurements are not representative of polymers’ compositions, monomer sequence, or topologies but are mostly affected by experimental procedures and conditions, such uncertainty will significantly sabotage the CGMD/ML analysis.

### 5.5 Future Directions

Ongoing experimental works attempt to generate and test the candidates selected from the ML works to validate the current inverse design from CGMD/ML models. Future computational studies might pay more attention to extending the chemical space and complexity (with varying chain lengths or more branched structures) in the polymer and polymer-like systems for more realistic applications, such as sequence-defined drug and nanoparticle designs, cell-penetrating peptides, copolymer designs with targeted self-assembly behaviors, etc. More efforts should be focused on parameterization or fine-tuning of CG potentials by including quantum-chemical or experimental data in specific applications to increase the reliability and consistency of this computational approach. Besides, polymer inverse design with multiple objectives is still one of the most challenging topics which can be leveraged in many practical applications, but still need more investigation on the model accuracy and transferability analysis (Jablonka et al., 2021). The inverse design of polymers in terms of both monomer chemical structures and monomer sequence is more appealing, as the chemical space will be significantly expanded from the hierarchical design. Moreover, only a few polymeric properties at the chain level (mostly focused on the value of the radius of gyration) have been explored in the current feed-forward property prediction and inverse design models. Hence, it is encouraged to consider diverse properties such as thermal, mechanical, optical, electronic properties, etc., in future research. Finally, while CGMD can capture the geometries of multiple-chain structures, characteristics of bulk material systems remain out of reach. It suggests developing methods to connect chain level properties with bulk assembly behaviors using broader critical parameter spaces such as polydispersity or assembly pathways, etc., to these materials (DeStefano et al., 2021).

## 6 Conclusion

**FIGURE 12:**
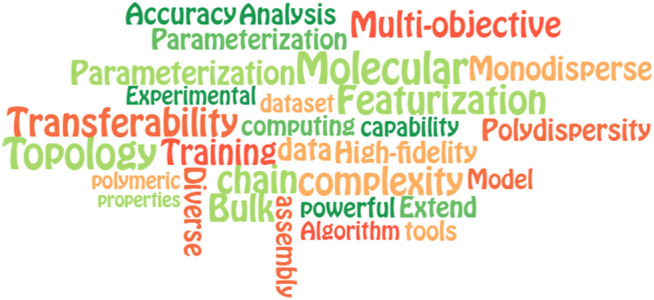
Keywords of challenges and future directions in this field.

In this review, we have surveyed the most recent applications of the hybridization of CGMD simulations with ML algorithms to solve the challenging problems in polymer science at the chain level, including configuration classification, feed-forward property prediction, and inverse molecular design. Throughout the manuscript, we also discussed some of the most powerful ML tools with basic knowledge and how to leverage these algorithms in further applications. Although CGMD/ML coupling has been demonstrated as a highly promising tool for polymer chain characterization and design, key challenges and issues remain to answer, as shown in Figure 12, opening many opportunities for more outstanding research in this field in the near future.

## Data Availability

The original contributions presented in the study are included in the article/Supplementary Material, further inquiries can be directed to the corresponding author.
